# A comprehensive landscape analysis of autophagy in cancer development and drug resistance

**DOI:** 10.3389/fimmu.2024.1412781

**Published:** 2024-08-26

**Authors:** Yue Li, Yang Yin, Tong Zhang, Jinhua Wang, Zeqi Guo, Yuyun Li, Ya Zhao, Ruihong Qin, Qian He

**Affiliations:** Department of Clinical Laboratories, The Second Affiliated Hospital of Xi’an Jiaotong University, Xi’an, China

**Keywords:** tumor resistance, pan-cancer, breast cancer, doxorubicin resistance, immune microenvironment

## Abstract

**Background:**

Autophagy plays important roles in cancer progression and therapeutic resistance, and the autophagy underlying the tumor pathogenesis and further mechanisms of chemoresistance emergence remains unknown.

**Methods:**

In this study, via the single-sample gene set enrichment analysis (ssGSEA) method, an autophagy 45-gene list was identified to evaluate samples’ autophagy activity, verified through six GEO datasets with a confirmed autophagy phenotype. It was further utilized to distinguish tumors into autophagy score-high and score-low subtypes, and analyze their transcriptome landscapes, including survival analysis, correlation analysis of autophagy- and resistance-related genes, biological functional enrichment, and immune- and hypoxia-related and genomic heterogeneity comparison, in TCGA pan-cancer datasets. Furthermore, we performed an analysis of autophagy status in breast cancer chemoresistance combined with multiple GEO datasets and *in vitro* experiments to validate the mechanisms of potential anticancer drugs for reversing chemoresistance, including CCK-8 cell viability assays, RT-qPCR, and immunofluorescence.

**Results:**

The 45-gene list was used to identify autophagy score-high and score-low subtypes and further analyze their multi-dimensional features. We demonstrated that cancer autophagy status correlated with significantly different prognoses, molecular alterations, biological process activations, immunocyte infiltrations, hypoxia statuses, and specific mutational processes. The autophagy score-low subtype displayed a more favorable prognosis compared with the score-high subtype, associated with their immune-activated features, manifested as high immunocyte infiltration, including high CD8+T, Tfh, Treg, NK cells, and tumor-associated macrophages M1/M2. The autophagy score-low subtype also showed a high hypoxia score, and hypoxic tumors showed a significantly differential prognosis in different autophagy statuses. Therefore, “double-edged” cell fates triggered by autophagy might be closely correlated with the immune microenvironment and hypoxia induction. Results demonstrated that dysregulated autophagy was involved in many cancers and their therapeutic resistance and that the autophagy was induced by the resistance-reversing drug response, in five breast cancer GEO datasets and validated by *in vitro* experiments. *In vitro*, dihydroartemisinin and artesunate could reverse breast cancer doxorubicin resistance, through inducing autophagy via upregulating LC3B and ATG7.

**Conclusion:**

Our study provided a comprehensive landscape of the autophagy-related molecular and tumor microenvironment patterns for cancer progression and resistance, and highlighted the promising potential of drug-induced autophagy in the activation of drug sensitivity and reversal of resistance.

## 1 Introduction

Autophagy, a type II programmed cell death, plays important roles in cancer progression, metastasis, and multidrug resistance (MDR) frequently followed by long-term cancer therapy. Although most patients with cancer initially responded quite well to the tumor treatment including chemotherapy, tumor metastasis and resistance to therapeutic drugs still inevitably occurred, leading to refractory cancer and tumor recurrence. Recently, autophagy, as a double-edged sword in cancer development and drug resistance, has been extensively concerned. Because of this “autophagy double-edged sword” in cancer development, autophagy also displays both promoting and inhibitory effects on tumor resistance and even MDR. On the one hand, it participates in the development of tumor resistance and protects cancer cells from chemotherapeutics; on the other hand, it kills MDR cancer cells in which apoptosis pathways are inactive. Autophagy induced by anticancer drugs could also activate apoptosis signaling pathways in cancers, facilitating MDR reversal (1).

Doxorubicin [DOX, also known as adriamycin (ADM)], a kind of DNA topoisomerase II inhibitor, belongs to an anthracycline anticancer drug family. However, the emergence of resistance after long-term DOX has become a major hindrance toward the effective treatment of a wide variety of cancer types (2). Chemoresistance is a critical risk problem for cancer treatment, including breast cancer. However, the mechanisms behind the emergence of chemoresistance remains unknown. Currently, the role of autophagy in cancer chemoresistance (DOX and so on) and the mechanisms involved have become one of the areas of intense investigation. The autophagy’s dual role leads to the significant specificity and contradictoriness in its association with tumor resistance, with varying outcomes across different research backgrounds, tumor types, and cell types. Many research teams indicated that the upregulation of many molecules acted as a key driver to the chemotherapy resistance via induced autophagy, for example, SH3BGRL [the study of Zhang et al. (3)] and heparinase [Anna Shteingauz et al. (4)]; in fact, these reflected the mainstream viewpoints that the increased resistance to various anticancer therapies could be associated with upregulation of autophagy. Thus, more and more preclinical data are being obtained on reversing resistance through modulation of autophagy, especially the monotherapy of autophagy inhibitors (chloroquine) or their combination with chemotherapeutic regimens to overcome chemoresistance, as one of the promising therapeutic strategies. However, more importantly, the autophagy in tumor resistance development remains an unknown and divisive issue. We also noticed some conflicting results; for example, Wu et al. reported that ADRB2 signaling inhibited autophagy, resulting in HIF1α stabilization and enhancing the resistance of hepatocellular cancer to sorafenib, and simultaneously, blocking ADRB2 could trigger the downregulation of HIF1α and further led to the reduction in glucose uptake and glycolysis, and improved antitumor effects (5). More emerging lines of evidence suggest the importance of focusing on the relationship between autophagy and the tumor microenvironment (TME). We thought that autophagy, that “double-edged” role causing different cell fates, might be related to the TME (immune response and hypoxia induction) and metabolic reprogramming that intersect with it. Meanwhile, different studies also support the paradoxical viewpoints about reversing chemoresistance; for example, some researchers indicated the inhibition of autophagy that could eliminate drug resistance (6–8), and others reported the induced autophagy for reversing resistance and enhancing chemotherapeutics sensitivity (9–11).

Therefore, our study aimed to reveal the influence of autophagy status on tumor progression and prognosis, especially the comprehensive analysis of autophagy-related molecules and the TME, and to further explore the mechanisms of autophagy regulation in relation to breast cancer chemoresistance (mainly DOX) and experimentally validate this phenomenon of several potential anticancer drugs with the effects of reversing DOX resistance *in vitro*. Through this comprehensive landscape of autophagy-related molecular alterations, our study will provide in-depth and novel insights into the pathological autophagy process and the future MDR tumor strategy by means of autophagy-targeting therapies.

## 2 Methods

### 2.1 Data source

**Figure 1:**
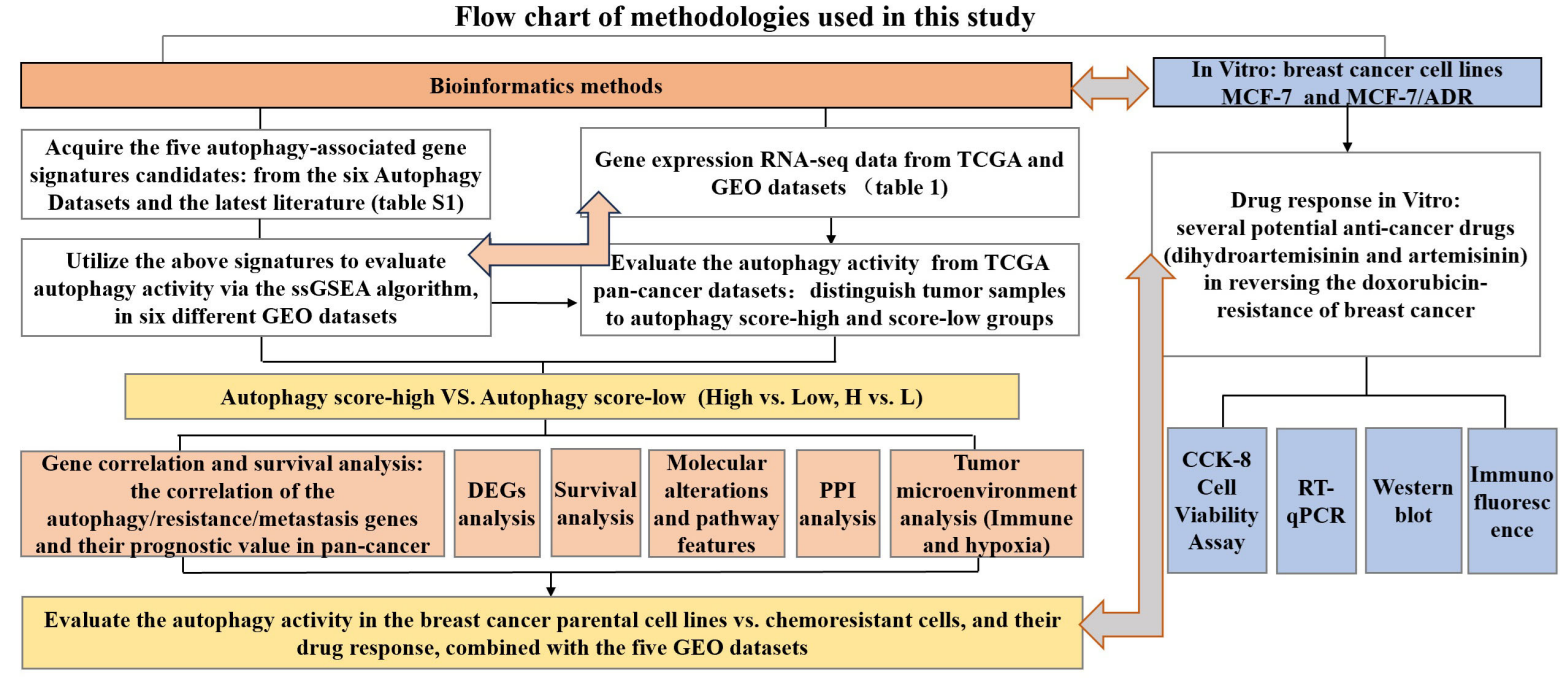
Flowchart of methodologies used in this study.

Gene expression datasets in our study and corresponding clinical information were obtained from The Cancer Genome Atlas (TCGA) and Gene-Expression Omnibus (GEO) databases (**Table 1**). The format of TCGA mRNA-seq data used in our study is HTseq-FPKM, which was the gene expression matrix obtained by standardizing the original read count values; thus, the data can be comparable between samples. We downloaded the series matrix of GEO datasets that has performed the quaintly normalization and background correction by RMA algorithm. The datasets used in this study did not require merging and all were used as independent verification sets. The Hallmark gene set of autophagy was acquired from the Molecular Signatures Database (MsigDB, https://www.gsea-msigdb.org/gsea/msigdb/). We also searched the canonical autophagy database Human Autophagy Database (HADb, http://www.autophagy.lu/index.html), and the 222-gene list was utilized for autophagy signature clustering candidates. Furthermore, we selected the overlapping autophagy-related gene sets from six databases (MSigDB, HADB, HAMDB, ncRDeathDB, THANATOS, and Autophagy) and the latest literature. The flowchart of methodologies used in our study is shown in **Figure 1**.

**Table 1:** Gene expression datasets and the main subjects used in this study.

Data source	Description	Main subjects
GEO databases	With the confirmed autophagy phenotypes	
GSE137359 (*N* = 8) (12)	Chidamide reverses the B-cell lymphoma chemoresistance by inducing autophagy	Low autophagy: rituximab-resistant/sensitive B cells High autophagy: chidamide-treated cells
GSE129203 (*N* = 8) (13)	GSK343, a potent autophagy inducer, inhibits hepatocellular carcinoma cells	Low: HepG2/PLC5 control cells High: GSK343-induced cells
GSE106175 (*N* = 6) (14)	Suberanilohydroxamic acid (SAHA) with the effects of lysosomal and autophagy activation	Low: control HeLa cells High: SAHA-treated HeLa cells
GSE46374 (*N* = 4) (15)	Atorvastatin inhibits prostate cancer proliferation by inducing autophagy	High: LNCaP/PC3 control cells Low: atorvastatin-treated cells
GSE185153 (*N* = 6)		High: HEK293 control cells Low: autophagy-deficient ATG7−/− HEK293
GSE31397 (*N* = 6)	miR-101-inhibited autophagy can sensitize breast cancer to 4-hydro-xytamoxifen-mediated cell death	High: MCF-7 control cells Low: miR-101 transfection cells
TCGA database	Exploring the relationships of autophagy and resistance in various cancer	
TCGA Pan-cancer	The standardized Pan-cancer dataset (*N* = 10,535) from the UCSC database (https://xenabrowser.net/)	Gene correlation analysis, based on the RNA-seq expression data of different genes in various cancer samples’ survival analysis, combined with its prognostic information
TCGA-BRCA	Breast cancer (*N* = 1,034)	
TCGA-PRAD	Bladder cancer (*N* = 396)	
TCGA-BLCA	Prostate cancer (*N* = 491)	
TCGA-STAD	Gastric cancer (*N* = 336)	
GEO databases	With the confirmed chemoresistance phenotypes	
GSE163361 (*N* = 84) (16)	Inflammatory breast cancer (SUM149 and FCIBC02 parental, and their paclitaxel-resistant, or doxorubicin-resistant cell lines), in biological triplicates	FCIBC02/SUM149 parental vs. paclitaxel-resistant vs. doxorubicin-resistant cells FCIBC02/SUM149 parental vs. drug-treated (paclitaxel or doxorubicin) FCIBC02/SUM149 chemoresistant cells vs. drug-treated (same concentration)
GSE54326 (*N* = 24) (17)	Breast cancer MDA-MB-231, MCF7, SKBR3, and ZR-75-1 parental and epirubicin-resistant cell lines	Parental vs. epirubicin-resistant, in biological triplicates
GSE125187 (*N* = 9) (18)	Breast cancer MCF-7 and their anthracycline-resistant cells	MCF-7 vs. doxorubicin-resistant vs. epirubicin-resistant cells
GSE155478 (*N* = 6) (19)		MCF-7 vs. doxorubicin-resistant
GSE202536 (*N* = 15)	Triple-negative breast cancer CAL51 and doxorubicin-resistant	CAL51 vs. doxorubicin-resistant CAL51/CAL51 doxorubicin-resistant cell vs. doxorubicin-treated (same concentration)

### 2.2 Identification of autophagy-associated gene signatures to evaluate autophagy status

In our study, the single-sample gene set enrichment analysis (ssGSEA) algorithm was applied to estimate the abundance of gene signatures for autophagy. ssGSEA was a computational method based on gene set enrichment analysis (20), similar to the commonly used GSEA method, is used to evaluate the degree of enrichment of specific functional gene sets in individual gene expression data, and is currently being used in many studies (21–23). Briefly, sort the expression of all genes in the sample to obtain their rank among all genes, and based on the input gene signature, sort and normalize the gene expression, to use the empirical cumulative distribution function of the genes in this signature to generate enrichment scores; shuffle the gene order and recalculate the enrichment score, repeating thousands of times to obtain the enrichment score of gene signature based on the distribution of gene enrichment scores. The higher the ssGSEA score, the more enriched this gene signature was in the sample. The ssGSEA results were analyzed using the R package “GSVA” to identify the overall expression characteristics of the autophagy gene set, and we imported dataset and gene lists and set parameters as “method=‘ssgsea’, kcdf=‘Gaussian’” in the use of the package “GSVA”. We attempted to evaluate the ability of the above five autophagy-associated gene signatures to distinguish the autophagy status among different samples. Six independent GEO datasets with known autophagy status (GSE137359, GSE129203, GSE106175, GSE46374, GSE185153, and GSE31397), involving different disease models wherein they could be divided into an autophagy-high and an autophagy-low group in any RNA-req dataset, were enrolled for the validation of the identification ability defined by the autophagy gene-signature-based ssGSEA score of each sample for autophagy activity.

Moreover, RNA-seq data from multiple GEO datasets with confirmed autophagic phenotypes were analyzed to identify differentially expressed genes (DEGs) of the autophagy-high group compared to the autophagy-low group, by using the limma package as the DEG method, and |log2 fold change (FC)| >1 and *p*-value <0.05 as the criteria to analyze a significant difference. In the GSE31397 dataset, we did not find any significant DEGs. Meanwhile, we also analyzed the gene expression of other molecules associated with tumor resistance (ATP binding cassette transporters: ABCA2, ABCA3, ABCB1, ABCB5, ABCB6, ABCB11, ABCC1, ABCC2, ABCC3, ABCC4, ABCC5, ABCC10, ABCC11, ABCF2, and ABCG2) (24). This transcriptomic analysis of the therapy resistance and autophagy landscapes in the autophagy-high group of multiple GEO datasets from a wide range of cancers could identify the close associations of autophagy activity and resistance.

### 2.3 Survival analysis

Furthermore, we analyzed the prognostic value of the autophagy-associated genes (*ULK2*, *ULK1*, *CDKN1A*, *CAMKK2*, *ATG7*, and G*ABARAPL1/2*) and resistance- or metastasis-associated genes (*ABCA2*, *ABCA3*, *ABCB1*, *ABCC3*, *ABCG1*, *ABCG2*, *ABCG4, ABCF2*; *MMP9*, *SNAI1*, *SNAI2*, and *SNAI3*) for multiple cancers, based on the TCGA pan-cancer database. The overall survival (OS) of patients with cancer with a follow-up time greater than 30 days was analyzed using the Kaplan–Meier method, based on the optimal cutoff value of the mRNA gene expression level where patients were divided into high and low expression, so as to analyze the prognostic value of the corresponding genes across 39 types of cancer. Survival analysis was considered significant based on log rank test *p* < 0.05.

### 2.4 Gene correlation

The TIMER web server (https://cistrome.shinyapps.io/timer/) is a comprehensive resource for systematical analysis of TCGA data. TIMER could explore the correlations between genes in multiple cancers.

### 2.5 Identification of differentially expressed genes

EdgeR, an R package, was used to identify the DEGs of RNA-Seq count data from TCGA cancer datasets. DEGs were corrected by FDR adjustment and considered significant following the criterion: |log2 fold change (FC)| >1; both the *p*-value and FDR < 0.05. In addition, GEO datasets were downloaded to identify the important genes, by using the limma data processing package as the DEG method and *p* < 0.05 as the criterion to analyze a significant difference. Based on the TCGA-BRCA dataset, we performed the DEG analysis and used DEGs between the autophagy score-high and score-low patients with breast cancer to conduct further analysis, including functional annotation, pathway enrichment, and protein–protein interaction (PPI) analysis. PPI network was constructed to extract hub genes through Cytoscape software 3.10.1, based on the STRING interactome (https://string-db.org/).

### 2.6 Biological function enrichment between the autophagy score-high and score-low groups

Module biological function was determined using g:Profiler (https://biit.cs.ut.ee/gprofiler) to identify enrichment for biological process in Gene Ontology (GO) and Kyoto Encyclopedia of Genes and Genomes (KEGG) pathways. GO consisted of biological process (BP), cellular component (CC), and molecular function (MF) (25). Enriched GO terms and KEGG pathways were considered to be meaning according to the criterion of adjusted *p* < 0.05 and selected top five when there were more than five records. In our study, we analyzed the biological processes and pathway enrichment of the DEGs between the score-high and score-low groups of breast cancer, based on the TCGA-BRCA dataset.

### 2.7 Tumor microenvironment analysis

After selecting the samples, we extracted the RNA-seq expression matrix from the samples and then each sample’s immune-stromal component in TME was calculated using the ESTIMATE algorithm performed in R language version 4.3.1: the immune infiltration (ImmuneScore), overall stromal content (StromalScore), and the combined (ESTIMATEScore). ImmuneScore, StromalScore, and ESTIMATEScore all displayed positive correlations with the ratio of immune, stromal, and the sum of the two components in TME; thus, higher scores indicated larger ratios of the respective component (26). Meanwhile, some bioinformatics methods using deconvolution-based analysis are supported to estimate the infiltrating immune cell proportion and score the immune microenvironment. The CIBERSORT method supports the characterization of cell composition and score estimation of 22 immune cells, according to different tissues from their gene expression profiles, which was utilized in a large amount of research (27). Therefore, we estimated the immune, stromal components in the TME of the autophagy score-high and score-low tumor tissues, and then performed the infiltrating immune cell proportion comparison between score-high and score-low breast cancer tissues, using the bioinformatics tool CIBERSORT.

### 2.8 Hypoxia score and tumor genomic heterogeneity comparison in autophagy score-high and score-low subtypes

Furthermore, our study analyzed the hypoxia score between the autophagy score-high and score-low group. Hypoxia score, using the hypoxia gene mRNA-based signatures constructed and validated in the previous literatures, included Winter score (Winter et al.), Buffa score (Buffa et al.), and Ragnum score (Ragnum et al.) (28–31). This was calculated as follows: patients with the top 50% of mRNA abundance data for each gene were given a score of +1; otherwise, the bottom 50% were −1. This process is repeated to calculate the total score. High scores defined the hypoxic tumor and low scores were indicative of normoxia. Tumor genomic heterogeneity analysis, including tumor mutational burden (TMB), microsatellite instability (MSI), and gene mutation landscape in all BC types were analyzed using the maftools R package with TCGA mutation data and online platforms for TCGA patient data based on R including the cBioPortal database (https://www.cbioportal.org/) and the SangerBox database (http://www.sangerbox.com/).

### 2.9 Cell culture

In our study, we used the breast cancer cell line that our team continuously used, MCF-7 cells (32). MCF-7 cells were one of the representative breast cancer cell lines, with positive estrogen receptor, positive progesterone receptor, and negative human epithelial receptor 2, and classified as luminal A subtype (33). We further successfully constructed the MCF-7 DOX-resistant cell line, MCF-7/ADM. The cells were cultured in DMEM medium, with 10% fetal calf serum (Sijiqing, ZhejiangTianhang Biotechnology Co. Ltd., China), 100 g/ml streptomycin, and 100 U/ml penicillin at 37°C with 5% CO_2_ in a humidified atmosphere.

### 2.10 Cell viability assay

Cell viability was measured by the CCK-8 assay. A total of 4×10^4^/ml MCF-7 or MCF-7/ADM cells were cultured in 96-well plates with four repeated holes per group and incubated overnight. As a continuation of the previous research (32), the cells were treated with different concentrations of dihydroartemisinin (DHA, MedChemExpress, MCE, USA, CAS: 71939-50-9) or artesunate (ART, MCE, CAS: 88495-63-0) for 48 h, including 5, 10, 20, and 40 μmol/L (μM) ART; 10, 20, 40, and 80 μmol/L (μM) DHA; and different concentrations of DOX hydrochloride (ADM, MCE, CAS: 25316-40-9). Then, 10 μl of CCK-8 (Genview, USA) was added and incubated at 37°C for 2 h. The optical density (OD) was measured on the ELISA plate reader (Tecan, Switzerland) at 450 nm. The measured values were analyzed by GraphPad Prism 7 and half-maximal inhibitory concentration (IC_50_) was calculated. The team successfully cultivated breast cancer MCF-7 cells and DOX-resistant MCF-7/ADM cells, and MCF-7/ADM cells were strongly resistant to DOX (drug resistance multiple >12).

### 2.11 Quantitative real-time polymerase chain reaction, immunofluorescence, and Western blot

MCF-7/ADM cells were cultured in medium with DHA for 48 h, and total RNA was extracted and reverse-transcribed using TRIzol reagent (Thermo Fisher, USA) and the PrimeScriptTM RT Reagent kit (TaKaRa, China). The Takara real-time fluorescent quantitative PCR system was used to detect the mRNA levels of *LC3B* and *ATG7* (qPCR supermix, TransGen Biotech, China). The PCR conditions were as follows: pre-denaturation at 95°C for 30 s, then 40 cycles of denaturation at 95°C for 5 s and annealing at 60°C for 30 s. The mRNAs were normalized with GAPDH and quantified via the 2^−ΔΔCT^ method. All primers were shown as follows: ATG7-forward, ATGATCCCTGTAACTTAGCCCA; ATG7-reverse, CACGGAAGCAAACAACTTCAAC; MAP1LC3B-forward, CACTGCTCTGTCTTGTGTAGGTTG; MAP1LC3B-reverse, TCGTTGTGCCTTTATTAGTGCATC; GAPDH-forward, GGAGCGAGATCCCTCCAAAAT; GAPDH- reverse, GGCTGTTGTCATACTTCTCATGG.

MCF-7/ADM cells were cultured in medium with 2 μM ADM, 20 μM ART/DHA, or 1 μM ADM +10 μM ART for 48 h, and then underwent the following steps: fixed with 100% methanol for 5 min, permeabilized in 0.1% Triton X-100 for 5 min, and then blocked with QuickBlockTM Blocking Buffer (Beyotime, China) for 15 min after three washes with washing liquid. The cells were incubated with anti-ABCG2, -ATG7, and -LC3B antibodies overnight at 4°C and Rabbit IgG (Alexa Fluor 488, Beyotime, P0176, China). The cells were counterstained with DAPI and then observed and photographed with 200× magnification using an inverted fluorescence microscope (Nikon, Japan). The results were quantified using the ImageJ software after determination of regions of interest and thresholds, represented by mean fluorescence intensity (34).

Total protein in the treated MCF-7/ADM cells was extracted with RIPA cell lysis (HAT, Xi’an, China) containing protease and phosphatase inhibitors and was measured using the BCA Protein Assay Kit (wanleibio, ShenYang, China). Protein samples (20 μg) were electrodeposited using a 4%–20% gradient polyacrylamide gel electrophoresis (PAGE) gel (FuturePAGETM prefabrication electrophoresis gel, ACE Biotechnology, China) in Tris-MES-SDS running buffer, transferred to a polyvinylidene fluoride (PVDF) membrane (Millipore, German), and then blocked with QuickBlock blocking solution (Beyotime, China) for 15 min. The membrane was incubated overnight at 4°C with primary antibodies against ATG7 (T57051, abmart, China) and GAPDH (ab8245, abcam), and then hatched with HRP-labeled goat anti-rabbit/mouse IgG for 2 h. Afterwards, an enhanced chemiluminescence reagent and an imaging system (Jiapeng, Shanghai, China) were utilized to detect the protein bands. Visualized band intensity was quantitatively analyzed using photoshop CS6 software, with GAPDH as the internal standard.

### 2.12 Statistical analysis

Statistical analysis was all performed with GraphPad Prism 9 and SPSS version 21.0. The results of two groups were analyzed by Student’s *t*-test, the results of three or more groups were determined by variance analysis, and further difference between groups was analyzed by the Dunnett method.

## 3 Results

### 3.1 Validating the autophagy gene set-based signature for the identification of autophagic activity

**Figure 2:**
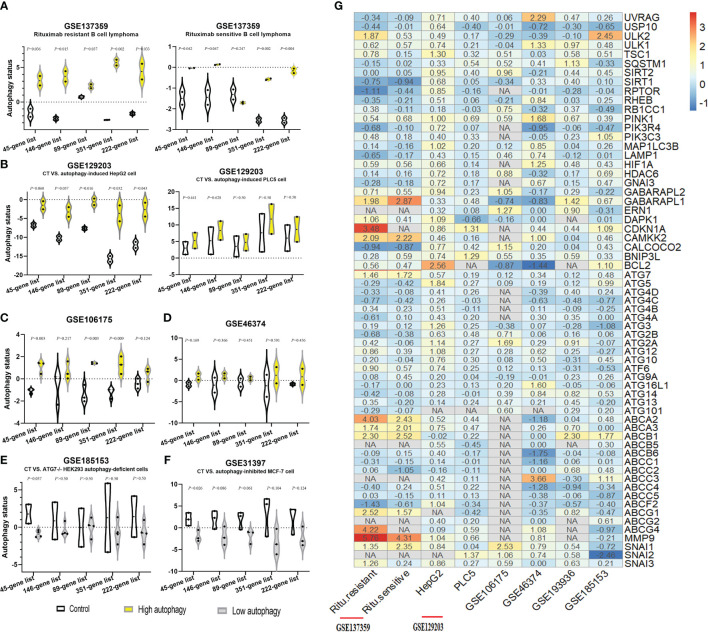
Identification of autophagy-associated gene signatures to evaluate autophagy status in different GEO datasets. **(A–F)** The autophagy status of six GEO datasets was evaluated by the ssGSEA scores of autophagy-associated gene signatures: the control group (CT, white), the autophagy score-high group (yellow), and the autophagy score-low group (gray). Two-sided Student’s *t*-test was used to assess the difference, *p* < 0.05. **(G)** Log2 fold change heatmap of the autophagy-high group, relative to the autophagy-low group, in six different GEO datasets. The ordinate is the gene name, and the number of the figures is the log2 fold change value of each gene, calculated as the mean gene expression in the autophagy-high group divided by the corresponding gene expression of the autophagy-low group and then take the log base 2. “NA” represents GEO datasets that did not contain these genes.

Firstly, we acquired the autophagy gene set-based signature candidates: from the MsigDB dataset, a 146-gene list associated with the positive regulation of autophagy and an 89-gene list associated with the negative regulation of autophagy and 351 autophagy-associated genes; from the HADb dataset, 222 autophagy gene sets (complete results in **Supplementary Table S1**). Moreover, we identified the overlapping autophagy-related gene sets from six databases (MSigDB, HADB, HAMDB, ncRDeathDB, THANATOS, and Autophagy) and the latest literature, which included 45 autophagy-associated genes (**Supplementary Table S1**). Furthermore, our study evaluated the ability of these autophagy-associated gene signatures to identify the autophagy status, in six different GEO datasets with known autophagy phenotype evidence (GSE137359, GSE129203, GSE106175, GSE46374, GSE185153, and GSE31397). Generally speaking, the autophagy scores of the above gene signatures can substantially distinguish the cell lines in autophagy-high status vs. autophagy-low status in at least four GEO datasets, especially the 45-gene signature based on its ssGSEA algorithm (**Figure 2**).

Moreover, compared with the autophagy-low group, we found that *ULK2*, *ULK1*, *CDKN1A*, *CAMKK2*, *ATG7*, and *LC3B* (*GABARAPL1/2*) were the major molecules with the significantly upregulated expression in the autophagy-high group. Interestingly, our study observed that the mRNA expression of some ATP binding cassette transporter family members significantly upregulated, for example, *ABCA2*, *ABCA3*, *ABCB1*, *ABCC3*, *ABCG1*, and *ABCG4*, simultaneously with the significant upregulation of metastasis-associated genes (*MMP9*, *SNAI1*, *SNAI2*, and *SNAI3*) as well in most datasets besides GSE185153 datasets (from human embryonic kidney). Based on previous research’s findings (1) and our results, we identified these autophagy-, resistance-, and metastasis-related genes. These findings could suggest the close associations between autophagy activity, drug resistance, and cancer metastasis, and provide ideas where elevated autophagic flux triggered the tendency of chemoresistance and metastasis.

### 3.2 Multi-omics autophagy-associated and resistance-associated effects across multiple cancer types and their associations

**Figure 3:**
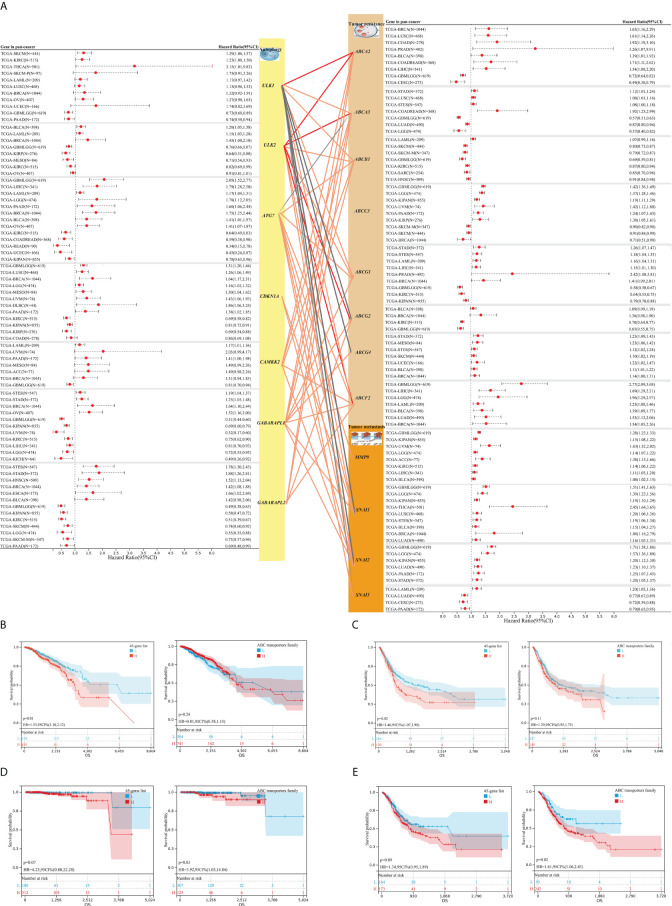
The associations between autophagy-associated and resistance/metastasis-associated molecules and their prognostic significance for TCGA pan-cancer. **(A)** Autophagy-associated genes (*ULK2*, *ULK1*, *CDKN1A*, *CAMKK2*, *ATG7*, and G*ABARAPL1/2*) and resistance- or metastasis-associated genes (*ABCA2*, *ABCA3*, *ABCB1*, *ABCC3*, *ABCG1*, *ABCG2*, *ABCG4, ABCF2*; *MMP9*, *SNAI1*, *SNAI2*, and *SNAI3*) showed the significantly prognostic value for overall survival (OS) across multiple tumors, based on TCGA pan-cancer datasets (complete survival data in **Supplementary Table S2**). The lines between gene modules represent their correlations (complete results in **Supplementary Table S3**). **(B–E)** The prognostic value of global molecular levels associated with the autophagy/resistance for breast cancer [45-gene list/ABC transporters family **(B)**)], bladder cancer **(C)**, prostate cancer **(D)**, and gastric cancer **(E)**, through the Kaplan–Meier method.

Furthermore, our study focused on the significant influence of autophagy-associated and resistance- or metastasis-associated genes on the prognosis of 44 cancers, and explored correlations between genes, based on TCGA pan-cancer datasets, to investigate the associations between autophagy and tumor resistance. We observed autophagy-associated genes (*ULK2*, *ULK1*, *CDKN1A*, *CAMKK2*, *ATG7*, and G*ABARAPL1/2*) and resistance- or metastasis-associated genes (*ABCA2*, *ABCA3*, *ABCB1*, *ABCC3*, *ABCG1*, *ABCG2*, *ABCG4, ABCF2*; *MMP9*, *SNAI1*, *SNAI2*, and *SNAI3*), showing the significant prognostic value for a wide range of tumors (shown in **Figure 3A**; complete survival data are provided in **Supplementary Table S2**), including breast cancer, bladder cancer, prostate cancer, and gastric cancer. In addition, the mRNA expression of these genes showed significant positive correlations in many tumors; meanwhile, there were also some genes that exhibited negative correlations between them (**Figure 3A**, complete results in **Supplementary Table S3**). To understand the global effects of molecular expressions associated with the autophagy/resistance process, we analyzed the ssGSEA gene enrichment fractions of these tumor samples from TCGA, and evaluated the prognostic influence of the autophagy/resistance score-high and score-low groups on breast cancer, bladder cancer, prostate cancer, and gastric cancer (**Figures 3B–E**). These results indicated the important influence of autophagy/resistance activity for the progression and prognosis of multiple cancers.

### 3.3 Associations between autophagy and mRNA/protein expression and signaling pathways

**Figure 4:**
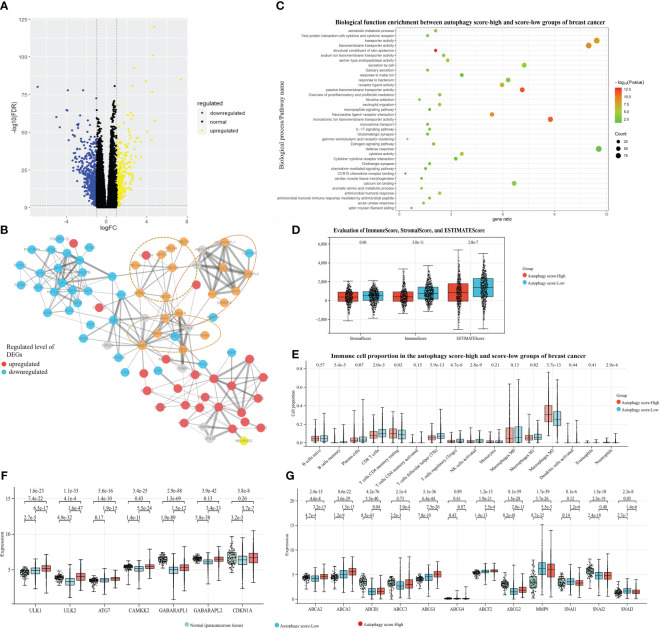
Associations between autophagy and mRNA/protein expression, signaling pathways, and tumor microenvironment (TME), with breast cancer data taken as an example. Based on the TCGA-BRCA dataset and their autophagy-associated ssGSEA score, our study identified the 856 DEGs between the autophagy score-high and score-low breast cancer patient groups: **(A)** The volcano plot of DEGs. **(B)** The protein–protein interaction network from DEGs and autophagy/chemoresistance/metastasis-associated key genes. **(C)** The functional pathway enrichment of DEGs. **(D)** The ImmuneScore, StromalScore, and ESTIMATEScore of two groups. **(E)** The comparison of immune cell proportion between two groups. **(F, G)** The gene expression analysis among the paracancerous tissue and the autophagy score-high and score-low breast cancer samples, presented in log2(TPM+1) format.

As shown in **Figure 2B**, we classified these breast cancer samples from the TCGA database into autophagy score-high and score-low groups (H, *N* = 427; L, *N* = 607), which exhibited the prognostic significance for breast cancer. The Kaplan–Meier survival curve suggested a higher risk of poor prognosis and death in the autophagy score-high group (HR = 1.53, 95% CI 1.10–2.12, *p* = 0.01). Furthermore, we identified the 856 DEGs, including the 292 up-expressed genes and 564 down-expressed genes between the above score-high and score-low groups. We found many significant molecular alterations associated with autophagy status (**Figures 4A, B**). Therefore, GO and pathway enrichment were carried out on these genes (**Figure 4C**). The GO enrichment analysis of DEGs was significantly enriched in defense response, response to metal ion, gamma-aminobutyric acid receptor clustering, cardiac muscle tissue morphogenesis, neuropeptide signaling pathway, chemokine-mediated signaling pathway, neuroactive ligand–receptor interaction, neutrophil migration, secretion by cell, acute-phase response, antimicrobial humoral immune response mediated by antimicrobial peptide, and so on. Similarly, the nine gene sets and the one gene set from KEGG or WikiPathways, respectively, were enriched in the autophagy score-high group compared with the score-low group. Among these pathway enrichments, gene sets associated with neuroactive ligand–receptor interaction, IL-17 signaling pathway, salivary secretion, cytokine–cytokine receptor interaction, viral protein interaction with cytokine and cytokine receptors, estrogen signaling pathway, cholinergic synapse, proinflammatory and profibrotic mediators, and so on were obviously enriched.

### 3.4 Associations between autophagy and the tumor microenvironment

The TME is essential in the development and progression of cancer. We further identified the mechanistic relationships of cancer autophagy status and the immune microenvironment. As shown in **Figure 4D**, the more immune and stromal components of TME were present in the autophagy score-low group. Comparison of immune cell proportions between the autophagy score-high and score-low groups (**Figure 4E**), also supported the idea that the autophagy status could influence the immune microenvironment including the infiltrating immune cell proportion; for instance, the increased proportions of infiltrating immune CD8+ T cells, Tfh, Treg cells, and NK cells were closely associated with the low autophagy status. Meanwhile, the score-low group showed the significantly high tumor-associated macrophages M1/M2 (*p* < 0.001). These results are consistent with many studies and our findings. Through the survival analysis of the overall TCGA-BRCA prognostic data, we found that the more immune components present in the TME displayed better prognosis (**Supplementary Figure S1**). Many studies demonstrated that the number, distribution, and polarization of macrophages played an important role in the progression of tumors (35–37); it would enhance the antitumorigenic response and cause increased survival by promoting macrophage polarization towards M1, the rise in the number of pro-inflammatory/antitumorigenic M1 macrophages, and the decrease in the anti-inflammatory/tumor-promoting M2 macrophages. These findings confirmed the close relationship between low autophagy status and a favorable prognosis and indicated the mechanisms of the elevated autophagy flux as tumor-protective autophagy via reducing antitumor immune response and enhancing the efflux of anticancer drugs (the ABC transporter mechanisms).

### 3.5 Associations between autophagy status and hypoxia-related signature

**Figure 5:**
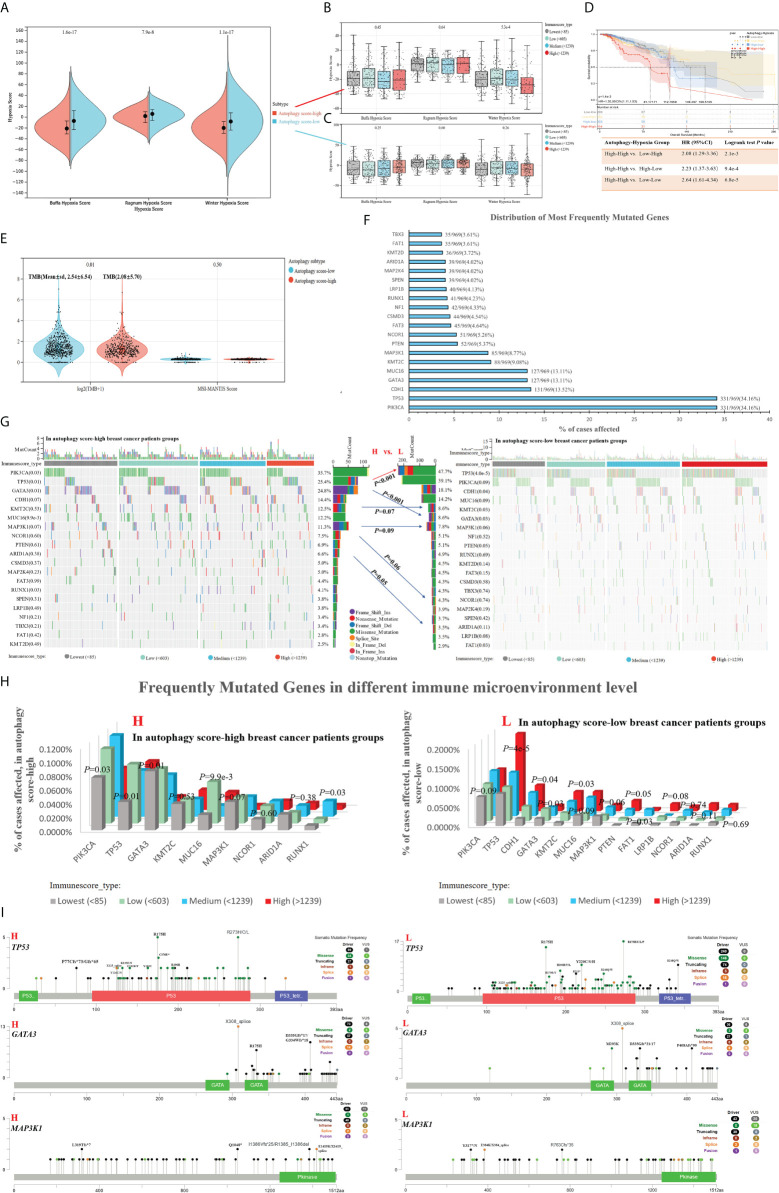
Associations between autophagy status and hypoxia score, tumor genomic heterogeneity comparison, with breast cancer data taken as an example. **(A)** Based on hypoxia-related signatures, Winter score, Buffa score, and Ragnum score, we analyzed the difference of hypoxia level in the autophagy score-high vs. score-low subtypes. **(B, C)** In different immune infiltration levels, the analysis of hypoxia score in the autophagy score-high **(B)** and score-low breast cancer subtypes **(C)**. **(D)** Survival analysis of individual autophagy status associated with hypoxia levels. **(E)** Tumor genomic heterogeneity analysis, TMB, and MSI, in the different autophagy status. **(F)** The top 15 most frequently mutated genes of breast cancer. **(G–I)** Gene mutation landscape in the autophagy score-high vs. score-low subtypes.

Previous works indicated that many tumors exhibited hypoxia, and hypoxic tumors often responded poorly to therapy and showed poorer prognosis (31). In our study, the autophagy score-low subtype displayed significantly activated hypoxia status, compared with the autophagy score-high subtype (**Figure 5A**). There was no significant difference in different immune cell infiltrations and hypoxic degrees (**Figures 5B, C**). In fact, the autophagy score-low subtype presented a more elevated hypoxia level and showed a certain degree of contradiction with its good prognosis. Therefore, we performed survival analysis and found a higher risk of death in the autophagy score-high/hypoxia score-high patients (H–H) than the autophagy score-low/hypoxia score-high patients (L–H) (HR = 2.08, 95% CI 1.29–3.36, *p* = 2.1e-3) and autophagy score-high/hypoxia score-low patients (H–L) (HR = 2.23, 95% CI 1.37–3.63, *p* = 9.4e-4) (**Figure 5D**). This suggested that the mechanisms of complex interactive processes caused differential cancer progression and prognosis, and different hypoxia levels interacting with cellular autophagy could trigger different cell fates.

### 3.6 Association between autophagy and molecular mutational alterations

TMB was a predictive biomarker of immunotherapy in multiple cancers, highly correlated with the efficacy of PD-1/PD-L1 inhibitors. The high TMB in tumors favors the infiltration of immune effector cells, and correlated with more strong antitumor immunotherapy responses. In our study, the autophagy score-low subtype displayed higher TMB (**Figure 5E**). We analyzed the top 15 most frequently mutated genes of breast cancer in the autophagy score-high vs. score-low subtypes (**Figures 5F, G**). Our study found the obvious changes in mutation frequency of *TP53* (25.4% vs. 47.7%, *p* = 6.7e-10, in High vs. Low patients with breast cancer), *GATA3* (24.8% vs. 8.6%, *p* = 1.6e-10), *KMT2C* (12.5% vs. 8.6%, *p* = 0.07), *MAP3K1*(11.3% vs. 7.8%, *p* = 0.09), *NCOR1*(7.5% vs. 4.3%, *p* = 0.06), and *ARID1A* (6.6% vs. 3.5%, *p* = 0.05), associated with breast cancer autophagy status (**Figure 5G**). Moreover, if the effects of the immune environment behind individual autophagy status is considered simultaneously, we found significant differences in the gene mutation frequency of *TP53*, *PIK3CA*, *GATA3*, *MUC16*, *MAP3K1*, and so on (**Figure 5H**). Among them, *TP53*, *GATA3*, and *MAP3K1* were overlapping genes, and we showed their specific mutation patterns (**Figure 5I**). At the gene level, driver mutations in *TP53*, *GATA3*, and *MAP3K1* are significantly enriched in the autophagy score-low group.

### 3.7 The role of autophagy in the development of tumor resistance

The previous results in this study indicated some potential associations between autophagy and tumor resistance. Considering the “autophagy paradox”, the dysregulation in autophagy has been suggested as a potential mechanism for chemoresistance, but there was still no consensus on whether autophagy promoted or inhibited chemoresistance (2). As shown in **Figures 3**, **4**, we could find the positive associations between autophagy and resistance mechanism, especially the significantly high expression of resistance-associated genes in the autophagy score-high group (**Figures 4F, G**), but we also noticed that the expression of some autophagy/resistance-associated genes was lower than that in paracancerous tissue. We would continue to investigate the autophagy status in known chemoresistant cancer cell lines.

**Figure 6:**
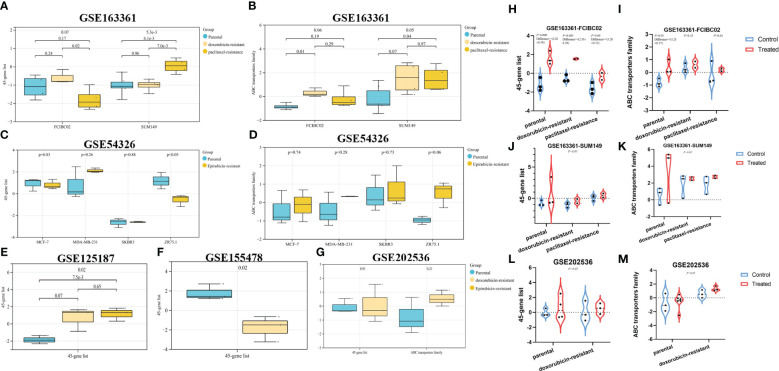
The evaluation of autophagy status in different GEO datasets. Based on the breast cancer GEO datasets and their autophagy/chemoresistance-associated ssGSEA score, our study mainly analyzed the autophagy status in parental vs. derived drug-resistant cells **(A–G)**, in parental/resistant cells vs. drug-treated cells **(H–M)**.

All breast cancer and drug-resistant GEO datasets were searched, to obtain a total of five expression data profiles (GSE163361, GSE54326, GSE125187, GSE155478 and GSE202536, **Table 1** and **Figures 6A–M**). Although limited by the sample size of each dataset, we could find that dysregulated autophagy was involved in the progression from drug-sensitive to drug-resistant cancer cells (**Figures 6A–G**). However, whether autophagy could promote or inhibit tumor resistance, thus exhibiting the “duality”, also depended on the type of cancer cell lines and cell nature states; for instance, in breast cancer MCF-7 DOX-resistant cells (MCF-7/ADM), there was a significant increase in autophagy in GSE125187 (**Figure 6E**), decreased autophagy in GSE155478 (**Figure 6F**), decreased autophagy in the ZR75.1 epirubicin-resistant cells based on GSE54326 (**Figures 6C, D**), and increased autophagy in SUM149 paclitaxel-resistant cells from GSE163361 (**Figures 6A, B**). Meanwhile, we noticed the more significant effect of chemotherapy drug-activated autophagy in the sensitive cancer cells than drug-resistant cells (in **Figures 6H–L**), which suggested the insensitivity to autophagy regulation in chemoresistant cancer.

### 3.8 Resistance reversing drug *in vitro*

**Figure 7:**
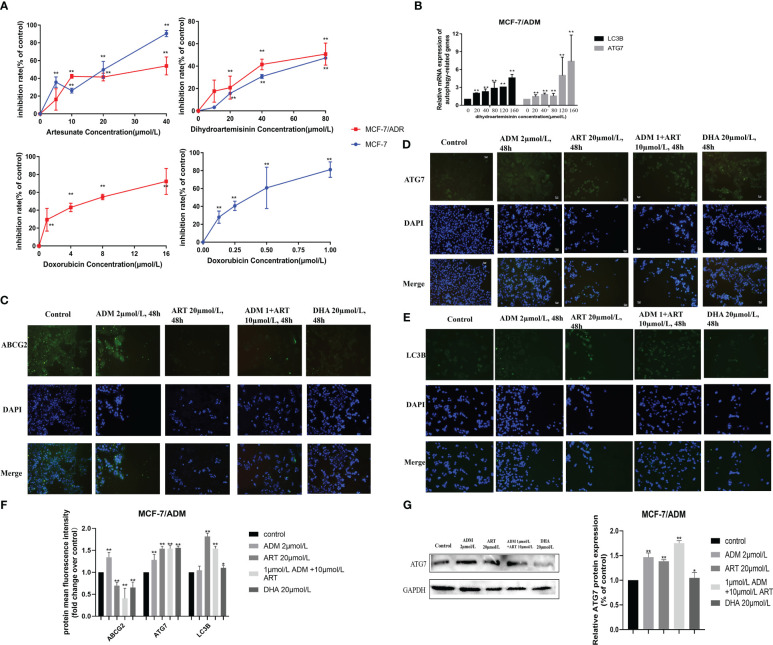
Several potential anticancer drugs (dihydroartemisinin and artesunate) in reversing the doxorubicin resistance of breast cancer *in vitro*. **(A)** The effect of dihydroartemisinin, artesunate, and doxorubicin on the proliferation inhibition rate of MCF-7 parental and its doxorubicin-resistant cell, MCF-7/ADM, was quantified by the CCK-8 method. Inhibition rate was calculated as the difference in optical density (OD) at 450 nm between the control and experimental group (OD_450_) divided by OD_450_ in the control group and then multiplied by 100%. **(B)** RT-qPCR analysis of the levels in cells, and corresponding quantification of the ATG7 and LC3B mRNA levels. **(C–E)** An immunofluorescence assay was used to detect ABCG2, ATG7, and LC3B proteins in MCF-7/ADM cells. MCF-7/ADM was untreated or treated with ADM (2 μmol/L)/ART (20μmol/L)/DHA (20 μmol/L) and combined group (ART+ADM) for 48 h Proteins were stained green, and nuclei were stained blue with 4,6-diamidino-2-phenylindole (DAPI). The images were captured at 200× magnification. **(F)** The immunofluorescence results (using mean fluorescence intensity) to reflect the changes in protein expression levels. **(G)** Western blot analysis of the ATG7 protein levels in MCF-7/ADM, and corresponding quantification of the ATG7 protein. *p < 0.05, **p < 0.01 vs. the control group.

Furthermore, we explored the drug response in reversing DOX resistance in breast cancer. Based on our previous research (32), we selected two drugs, DHA and ART, which could significantly inhibit the proliferation of breast cancer cell MCF-7 (**Figure 7A**), and found their potential to reverse DOX resistance in breast cancer. We generated isogenic MCF7 DOX-resistant breast cancer cell lines (MCF-7/ADM), which were insensitive to the same DOX concentration that could trigger the massive cell death of parental MCF-7 cells. DHA and ART could significantly inhibit the proliferation of MCF-7/ADM cells in a dose-dependent manner (**Figure 7A**). Moreover, we analyzed the autophagy status of MCF-7/ADM cells on different conditions by the expression analysis of autophagy-related molecules (LC3B and ATG7) via quantitative real-time polymerase chain reaction (RT-qPCR) and immunofluorescence, to confirm the activation of autophagy by DHA/ART treatment for the inhibition of breast cancer DOX-resistant cells (**Figures 7B, D, E**). As shown in **Figures 7C–G**, we could find that DHA and ART could upregulate the LC3B and ATG7 protein expression and inhibit ABCG2 protein expression, and the ART+ADM combined treatment group had a more significant effect compared with the control group. Therefore, DHA and ART played an important role in reversing DOX resistance in breast cancer, through activating the autophagy pathway via the regulation of LC3B and ATG7.

## 4 Discussion

Autophagy, a self-degradative system, plays important roles in cancer progression, therapeutic resistance, and the treatment of sensitive and MDR cancer. Undeniably, cytotoxic drugs including anthracyclines remain the first-line option for many cancer therapies, but the development of drug resistance has emerged as a major obstacle to effective treatment. Therefore, understanding the autophagy process is becoming increasingly important for preventing tumor recurrence and combating cancer therapeutic resistance. However, because of the dual role of autophagy in cancer progression and resistance, the autophagy underlying tumor pathogenesis and its related mechanisms that result in the emergence of chemoresistance remain complicated and contradictory.

There is evidence that autophagy prevents cancer during the early steps of carcinogenesis, but once transformed, these cells show enhanced autophagy capacity and use it to survive, grow, and facilitate metastasis (38, 39). In our study, based on the ssGSEA algorithm, several autophagy-associated gene signatures from six autophagy databases (MSigDB, HADB, HAMDB, ncRDeathDB, THANATOS, and Autophagy) were used to evaluate the autophagy activity of samples with confirmed autophagy phenotype, and a new 45-gene signature was identified with the good evaluation ability to distinguish samples between the autophagy score-high and autophagy score-low groups by validating in six different GEO datasets. Based on that, we could classify TCGA pan-cancer samples across 39 cancer types into autophagy score-high and score-low groups; taking breast cancer, bladder cancer, prostate cancer, and gastric cancer as an example, we demonstrated the important influence of autophagy activity and resistance-associated molecule activity for the prognosis of many tumors.

**Figure 8:**
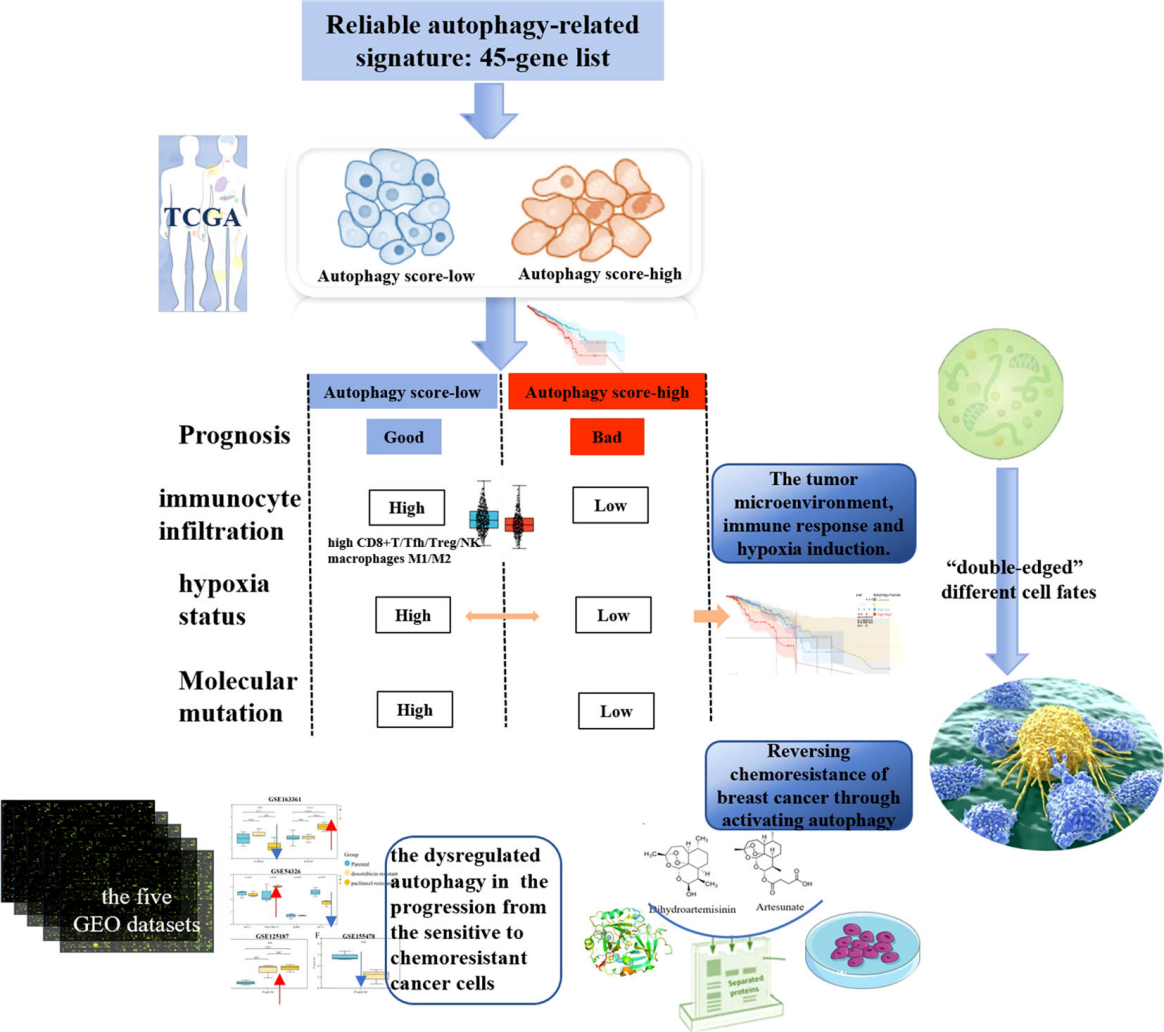
Dysregulated autophagy was involved in cancers and their therapeutic resistance.

Furthermore, we characterized the comprehensive autophagy-related multi-dimensional landscapes in the comparison of breast cancer autophagy score-high and score-low patients (**Figure 8**). Furthermore, our results demonstrated that cancer autophagy status correlated with significantly different prognosis, molecular alterations, biological processes activation, immunocyte infiltration, hypoxia status, specific mutational processes, and therapeutic response. The autophagy score-low subtype manifested as the more favorable prognosis, which might be caused by an abundant immune microenvironment and increased mutational load. The paradoxical roles of the autophagy on cancer, as inducer or tumor suppressor, might depend on different stages of cancer development. In fact, we believed that the favorable prognosis of autophagy score-low tumors was consistent with the existing understanding that autophagy prevented cancer development in the early stages, but later on enhanced autophagy and further facilitated cancer cells to survive, grow, and metastasize, which might be because most of these patients with cancer do not belong to the early stage when tumor samples are tested. Many studies indicated that this “double-edged” function did depend on the different stages of cancer development and is further determined by environmental conditions such as nutrient availability, immune system status, pathogenic conditions, and microenvironmental stress (40, 41). Interestingly, autophagy status showed different hypoxia levels, especially in the score-low subtype with a more elevated hypoxia level, and hypoxic tumors with significantly different prognosis in the autophagy score-low and score-high subtypes, which indicated that “double-edged” different cell fates triggered by autophagy might be closely related to the TME, immune response, and hypoxia induction.

The excessive proliferation characteristics of cancer cells led to a demand for oxygen. Under the activation of a hypoxia-inducible factor (HIF) in response to hypoxia conditions, it induced and promoted angiogenesis to provide oxygen, in order to promote previously stagnant tumor cell proliferation. This once again required oxygen supply, to further recreate the hypoxic microenvironment and HIF activation (42, 43). That is to say, the cross-talk between tumor progression and HIF activation is a never-ending phenomenon, which is further worsened by the involvement of autophagy, and some studies indicated the involvement of hypoxia-induced autophagy in cancer progression (40, 42, 44). Our results confirmed the poorer prognosis of the autophagy high-score hypoxic tumor compared with that of the autophagy high-score/hypoxia low-score tumors (High–High vs. High–Low, HR = 2.23, 95% CI: 1.37–3.63), in patients with breast cancer (**Figure 5D**, High–High vs. Low–High, HR = 2.08, 95% CI: 1.29–3.36). In autophagy low-score tumors, hypoxia scores did not significantly affect prognosis (**Figure 5D**). Therefore, we believed that the findings were consistent with some reports documenting that hypoxic tumors and autophagy-activated tumors had a higher degree of malignancy (45). Some studies have confirmed that targeting the hypoxic microenvironment or autophagy is helpful for antitumor therapy (46).

The current evidence confirmed that autophagy could promote immune evasion to inhibit antitumor immunity (47). Several GO and KEGG pathways associated with immune response (neuropeptide signaling pathway, chemokine-mediated signaling pathway, neutrophil migration, acute-phase response, and antimicrobial humoral immune response mediated by antimicrobial peptide) showed the differential enrichment. Notably, the autophagy score-low subtype manifested as the more favorable prognosis, which might be caused by their abundant immune microenvironment, including increased proportions of infiltrating immune CD8+ T cells, Tfh, Treg cells, and NK cells and high tumor-associated macrophages M1/M2, and increased mutational load (high TMB and gene mutation frequency). These findings suggested the mechanisms of the elevated autophagy activity as a tumor-protective process via reducing antitumor immune response. Some studies also indicated that the loss of autophagy by knockdown of Atg7 or chloroquine treatment caused accumulation of major histocompatibility complex (MHC) class I proteins on the tumor cell surface to recognize and target CD8 T cells (47). The inhibition of autophagy led to the infiltration and the transition of tumor-associated macrophages from M2 to M1 with antitumorigenic activity in melanoma (48). In colon cancer, the combination of anticancer agents 5-FU and chloroquine promoted the maturation of dendritic cells and caused the stimulation of CD8+ T-cell response (49). Therefore, the increased CD8+ T cells and macrophages M1/M2 associated with the low-autophagy activity might act as an important mechanism of the autophagy affecting tumor survival and prognosis, which suggests that the reason why autophagy leads to different cell fates might be closely related to its cross-talk with the TME.

This transcriptomic analysis of the autophagy landscapes in the score-high/low groups of multiple GEO datasets and TCGA pan-cancer datasets could also identify the close associations of autophagy activity and resistance across a wide range of cancers. In fact, the drug resistance to key anticancer agents in recent years has complicated the procedures for tumor therapy. DOX is considered as a potent anticancer agent in both early and advanced stages of cancer (50). Increasing lines of evidence show that chemotherapeutic regimens containing anthracyclines such as DOX are superior in cancer therapy compared to regimens lacking anthracyclines (51, 52). However, the resistance to DOX arises and develops owing to its frequent application, and has become a major barrier for cancer therapy.

Many recent studies have shown that dysregulated autophagy was known to be involved in chemoresistance of many cancer cells (53). Increased autophagy has been shown to promote or inhibit drug resistance depending, to a large extent, on the type of tumor involved and the nature of treatment-induced metabolic stress (1, 2, 54). On the one hand, some studies show that autophagy promotes cell survival and results in DOX resistance. Pan et al. revealed that increased autophagy and decreased apoptosis were found in DOX-resistant multiple myeloma RPMI8226/DOX cells (55). Jiang et al. pointed out that TBX15 overexpression can abrogate breast cancer DOX resistance by suppressing glycolysis and autophagy (53). These findings demonstrate that protective autophagy cause decreased sensitivity to DOX in cancer cells, leading to drug resistance and even MDR. On the other hand, some studies demonstrate that induction of autophagy helps activate cell death in a wide range of cancers, facilitating to overcome DOX resistance. For example, Aleksandra et al. found that hTERT downregulation attenuates resistance to DOX in BC, by the related mechanisms of the impairment of FAK-mediated adhesion and the induction of autophagy (9); RAD001, an autophagy activator, increased LC-3II expression and autophagosome formation in human papillary thyroid cancer (PTC), which resulted in an increased sensitivity to DOX (56); Atg5-deficient HeLa and breast cancer MDA-MB-231 cells showed obviously decreased sensitivity toward DOX (57); which demonstrates that autophagy confers enhanced sensitivity to DOX in cancer cells. In fact, there are still limited studies on DOX resistance and autophagy, especially those involving drugs that can reverse DOX resistance. Therefore, an in-depth understanding of the factors and mechanisms involved in DOX resistance will be of great significance for developing novel strategies to overcome these resistances in BC.

In our study, we further investigated the autophagy status in breast cancer chemoresistance, combined with multiple GEO datasets and experiments *in vitro* to validate the corresponding mechanisms of several potential anticancer drugs for reversing tumor resistance. In the transformation from breast cancer drug-sensitive to chemoresistant cells, limited by small sample size, whether autophagy could promote or inhibit tumor resistance, thus exhibiting the “duality”, and also depended on the type of cancer cell lines and specific cell states. In recent years, the cross-talk of the hypoxia microenvironment, immune microenvironment, and autophagy in the progression of tumor drug resistance attracted widespread attention. Many studies believed that the continuously reactivated HIF further enhanced tumor proliferation, metastasis, and resistance. Wei et al. reported that the hypoxia-induced autophagy via HIF1A-associated Beclin-1 caused cancer radioresistance (58). Feng et al. demonstrated that liensinine (novel anticancer agents) could overcome the resistance of colorectal cancer cells to oxaliplatin by suppressing HIF-1α levels to inhibit autophagy (59). Of course, increasing evidence has shown that the specific role of autophagy in tumorigenesis and resistance may be related to the specific stage and type, and exhibited the “duality”, which was consistent with our results. However, this remained an important limitation of this study. In the future, more rigorous molecular biological experiments *in vitro* and *in vivo* are required to explore their associations.

Survival through autophagy is a key reason enabling long-term tumor viability and eventual regrowth and tumor recurrence (60). For example, cisplatin-mediated chemoresistance played a pro-survival role via activating autophagy, simultaneously manifested as epithelial–mesenchymal transition (EMT) including upregulating vimentin in nasopharyngeal carcinoma (61). However, notably, here the protective autophagy induced by tumor cells was working, causing the abnormal expression of apoptosis-associated genes, which enabled the tumor to gain resistance to apoptosis, leading to drug resistance. Therefore, many studies also believed that autophagy could act as a cell death program when the apoptotic mechanism was deficient; thus, therapeutic induction of autophagic cell death through excessive stimulation of autophagy remained an important method for eliminating tumor cells (9, 56). In our study, another issue was the more obvious effect of chemotherapy drug-induced autophagy in the sensitive/parental cancer cells than drug-resistant cells, which might suggest the insensitivity to autophagy regulation in chemoresistant cancer. More importantly, utilizing DHA and ART, we further experimentally validate this finding. Our analysis revealed that autophagy induction via some potential drugs might sensitize cancer cells to anticancer drugs and reverse tumor resistance. DHA and ART could reverse DOX resistance in breast cancer, through activating the autophagy pathway via the regulation of LC3B and ATG7. Current results showed the feasibility of using autophagy-induced drugs to improve the efficiency of anticancer therapy.

## 5 Conclusion

Dysregulated autophagy was involved in many cancers and their therapeutic resistance, further leading to the significant differences in prognosis and therapeutic drug response of patients with cancer. Our findings indicated the important influence of autophagy/resistance activity in prognosis across 39 cancer types, and further characterized that the cancer autophagy status correlated with significantly different prognoses, molecular alterations, biological process activations, immunocyte infiltrations, hypoxia statuses, specific mutational processes, and therapeutic responses. The autophagy score-low subtype manifested as the more favorable prognosis, which might be caused by an abundant immune microenvironment and increased mutational load. Interestingly, hypoxic tumors have a significantly different prognosis in the autophagy score-low and score-high subtypes, which indicated that “double-edged” different cell fates triggered by autophagy might be closely related to the TME, immune response, and hypoxia induction. However, in the transformation of breast cancer parental to chemoresistant cells, whether autophagy could promote or inhibit tumor resistance, thus exhibiting the “duality”, also depended on the type of cancer cell lines and cell nature states, based on multiple GEO datasets. Generally speaking, our study provided a comprehensive landscape of the autophagy-related multi-dimensional alteration patterns for cancer progression and resistance, and highlighted the promising potential of drug-induced autophagy in the activation of drug sensitivity and reversal of resistance.

## Data Availability

The datasets presented in this study can be found in online repositories. The names of the repository/repositories and accession number(s) can be found in the article/**Supplementary Material**.
