# Supplementary material for: A comprehensive landscape analysis of autophagy in cancer development and drug resistance
Source: Front Immunol. 2024 Aug 26;15:1412781. doi: 10.3389/fimmu.2024.1412781 (PMC11381251; doi:10.3389/fimmu.2024.1412781)
Supplement: Supplementary file 2 [file DataSheet2.pdf]

Table S2 The survival analysis of hub genes in the TCGA Pancancer datasets

Gene in pan-cancer	OS	HR	Lower	Upper	Hazard Ratio(95%CI)
ULK1					
CancerCode	pvalue	HR	Lower	Upper	Hazard Ratio(95%CI)
TCGA-SKCM(N=444)	0.01	1.29	1.06	1.57	1.29(1.06,1.57)
TCGA-KIRC(N=515)	0.05	1.23	1.00	1.50	1.23(1.00,1.50)
TCGA-THCA(N=501)	0.05	3.15	1.01	9.83	3.15(1.01,9.83)
TCGA-SKCM-P(N=97)	0.09	1.73	0.91	3.26	1.73(0.91,3.26)
TCGA-LAML(N=209)	0.1	1.17	0.97	1.42	1.17(0.97,1.42)
TCGA-LUSC(N=468)	0.13	1.13	0.96	1.33	1.13(0.96,1.33)
TCGA-BRCA(N=1044)	0.13	1.32	0.92	1.91	1.32(0.92-1.91)
TCGA-OV(N=407)	0.07	1.27	0.98	1.65	1.27(0.98,1.65)
TCGA-UCEC(N=166)	0.14	1.74	0.82	3.69	1.74(0.82,3.69)
TCGA-GBMLGG(N=619	0.0019	0.73	0.60	0.89	0.73(0.60,0.89)
TCGA-PAAD(N=172)	0.01	0.74	0.58	0.94	0.74(0.58,0.94)
ULK2					
CancerCode	pvalue	HR	Lower	Upper	Hazard Ratio(95%CI)
TCGA-BLCA(N=398)	0.0093	1.20	1.05	1.38	1.20(1.05,1.38)
TCGA-LAML(N=209)	0.02	1.15	1.03	1.28	1.15(1.03,1.28)
TCGA-BRCA(N=1044)	0.05	1.45	1.00	2.10	1.45(1.00,2.10)
TCGA-GBMLGG(N=619	0.000098	0.76	0.66	0.87	0.76(0.66,0.87)
TCGA-KIRP(N=276)	0.00011	0.64	0.51	0.80	0.64(0.51,0.80)
TCGA-MESO(N=84)	0.01	0.71	0.54	0.93	0.71(0.54,0.93)
TCGA-KIRC(N=515)	0.04	0.82	0.69	0.99	0.82(0.69,0.99)
TCGA-OV(N=407)	0.08	0.91	0.81	1.01	0.91(0.81,1.01)
ATG7					
CancerCode	pvalue	HR	Lower	Upper	Hazard Ratio(95%CI)
TCGA-GBMLGG(N=619	4.1E-06	2.05	1.52	2.77	2.05(1.52,2.77)
TCGA-LIHC(N=341)	0.00069	1.79	1.28	2.50	1.79(1.28,2.50)
TCGA-KICH(N=64)	0.0045	12.81	2.07	79.34	12.81(2.07,79.34)
TCGA-LAML(N=209)	0.0086	1.17	1.04	1.31	1.17(1.04,1.31)
TCGA-LGG(N=474)	0.02	1.78	1.12	2.85	1.78(1.12,2.85)
TCGA-PAAD(N=172)	0.03	1.60	1.06	2.44	1.60(1.06,2.44)
TCGA-THYM(N=117)	0.05	6.48	0.96	43.58	6.48(0.96,43.58)
TCGA-BRCA(N=1044)	0.00095	1.75	1.25	2.44	1.75(1.25,2.44)
TCGA-BLCA(N=398)	0.04	1.41	1.01	1.97	1.41(1.01,1.97)
TCGA-OV(N=407)	0.01	1.41	1.07	1.87	1.41(1.07-1.87)
TCGA-KIRC(N=515)	0.00083	0.64	0.49	0.83	0.64(0.49,0.83)
TCGA-COADREAD(N=[illegible]	0.01	0.59	0.38	0.90	0.59(0.38,0.90)
TCGA-READ(N=90)	0.01	0.34	0.15	0.78	0.34(0.15,0.78)
TCGA-UCEC(N=166)	0.02	0.45	0.24	0.87	0.45(0.24,0.87)
TCGA-KIPAN(N=855)	0.02	0.78	0.63	0.96	0.78(0.63,0.96)
CDKN1A					
CancerCode	pvalue	HR	Lower	Upper	Hazard Ratio(95%CI)
TCGA-GBMLGG(N=619	4E-09	1.31	1.20	1.44	1.31(1.20,1.44)
TCGA-LUSC(N=468)	0.0089	1.26	1.06	1.49	1.26(1.06,1.49)
TCGA-BRCA(N=1044)	0.0036	1.64	1.17	2.31	1.64(1.17,2.31)
TCGA-LGG(N=474)	0.02	1.16	1.02	1.32	1.16(1.02,1.32)
TCGA-MESO(N=84)	0.02	1.30	1.04	1.62	1.30(1.04,1.62)
TCGA-UVM(N=74)	0.02	1.43	1.06	1.93	1.43(1.06,1.93)
TCGA-DLBC(N=44)	0.02	1.86	1.06	3.24	1.86(1.06,3.24)
TCGA-PAAD(N=172)	0.03	1.38	1.02	1.85	1.38(1.02,1.85)
TCGA-KIRC(N=515)	0.000038	0.69	0.58	0.82	0.69(0.58,0.82)
TCGA-KIPAN(N=855)	0.0003	0.81	0.72	0.91	0.81(0.72,0.91)
TCGA-KIRP(N=276)	0.003	0.69	0.54	0.88	0.69(0.54,0.88)
TCGA-COAD(N=278)	0.2	0.86	0.69	1.08	0.86(0.69,1.08)
CAMKK2					
CancerCode	pvalue	HR	Lower	Upper	Hazard Ratio(95%CI)

TCGA-LAML(N=209)	0.03	1.17	1.01	1.36	1.17(1.01,1.36)
TCGA-UVM(N=74)	0.05	2.03	0.99	4.17	2.03(0.99,4.17)
TCGA-PAAD(N=172)	0.05	1.41	1.00	1.98	1.41(1.00,1.98)
TCGA-MESO(N=84)	0.06	1.49	0.99	2.26	1.49(0.99,2.26)
TCGA-ACC(N=77)	0.06	1.49	0.98	2.24	1.49(0.98,2.24)
TCGA-BRCA(N=1044)	0.11	1.31	0.94	1.83	1.31(0.94,1.83)
TCGA-GBMLGG(N=619	0.0069	0.81	0.70	0.94	0.81(0.70,0.94)
TARGET-NB(N=151)	0.04	0.70	0.49	0.99	0.70(0.49,0.99)
GABARAPL1					
CancerCode	pvalue	HR	Lower	Upper	Hazard Ratio(95%CI)
TCGA-STES(N=547)	0.01	1.19	1.04	1.37	1.19(1.04,1.37)
TCGA-STAD(N=372)	0.01	1.25	1.05	1.48	1.25(1.05,1.48)
TCGA-BRCA(N=1044)	0.01	1.64	1.10	2.44	1.64(1.10,2.44)
TCGA-OV(N=407)	0.0024	1.52	1.16	2.00	1.52(1.16,2.00)
TCGA-GBMLGG(N=619	3.8E-16	0.51	0.44	0.60	0.51(0.44,0.60)
TCGA-KIPAN(N=855)	6.3E-08	0.69	0.60	0.79	0.69(0.60,0.79)
TCGA-UVM(N=74)	0.00041	0.32	0.17	0.60	0.32(0.17,0.60)
TCGA-KIRC(N=515)	0.0029	0.75	0.62	0.90	0.75(0.62,0.90)
TCGA-LIHC(N=341)	0.0033	0.81	0.70	0.93	0.81(0.70,0.93)
TCGA-LGG(N=474)	0.02	0.72	0.55	0.95	0.72(0.55,0.95)
TCGA-KICH(N=64)	0.02	0.49	0.26	0.92	0.49(0.26,0.92)
GABARAPL2					
CancerCode	pvalue	HR	Lower	Upper	Hazard Ratio(95%CI)
TARGET-LAML(N=142	0.000055	1.61	1.28	2.04	1.61(1.28,2.04)
TCGA-STES(N=547)	0.0003	1.78	1.30	2.43	1.78(1.30,2.43)
TCGA-STAD(N=372)	0.002	1.88	1.26	2.81	1.88(1.26,2.81)
TCGA-HNSC(N=509)	0.0052	1.52	1.13	2.04	1.52(1.13,2.04)
TCGA-BRCA(N=1044)	0.01	1.42	1.08	1.88	1.42(1.08,1.88)
TCGA-PCPG(N=170)	0.02	21.06	1.69	262.81	21.06(1.69,262.81)
TCGA-ESCA(N=175)	0.04	1.66	1.02	2.69	1.66(1.02,2.69)
TCGA-BLCA(N=398)	0.06	1.42	0.98	2.06	1.42(0.98,2.06)
TCGA-GBMLGG(N=619	6.6E-08	0.49	0.38	0.63	0.49(0.38,0.63)
TCGA-KIPAN(N=855)	6.9E-07	0.58	0.47	0.72	0.58(0.47,0.72)
TCGA-KIRC(N=515)	7.8E-07	0.51	0.39	0.67	0.51(0.39,0.67)
TCGA-SKCM(N=444)	0.0054	0.74	0.60	0.92	0.74(0.60,0.92)
TCGA-LGG(N=474)	0.01	0.55	0.35	0.88	0.55(0.35,0.88)
TCGA-SKCM-M(N=347	0.01	0.73	0.57	0.94	0.73(0.57,0.94)
TCGA-PAAD(N=172)	0.05	0.69	0.48	0.99	0.69(0.48,0.99)
ABCA2					
CancerCode	pvalue	HR	Lower	Upper	Hazard Ratio(95%CI)
TCGA-BRCA(N=1044)	0.0048	1.63	1.16	2.29	1.63(1.16,2.29)
TCGA-LUSC(N=468)	0.0062	1.61	1.14	2.26	1.61(1.14,2.26)
TCGA-COAD(N=278)	0.0068	1.92	1.19	3.10	1.92(1.19,3.10)
TCGA-PRAD(N=492)	0.04	3.26	1.07	9.91	3.26(1.07,9.91)
TCGA-BLCA(N=398)	0.04	1.39	1.01	1.92	1.39(1.01,1.92)
TCGA-COADREAD(N=	0.01	1.71	1.11	2.62	1.71(1.11,2.62)
TCGA-LIHC(N=341)	0.02	1.54	1.08	2.20	1.54(1.08,2.20)
TCGA-GBMLGG(N=619	2.9E-07	0.72	0.64	0.82	0.72(0.64,0.82)
TCGA-PAAD(N=172)	0.0064	0.73	0.58	0.91	0.73(0.58,0.91)
TCGA-KIPAN(N=855)	0.02	0.84	0.73	0.98	0.84(0.73,0.98)
TCGA-CESC(N=273)	0.0027	0.49	0.30	0.79	0.49(0.30,0.79)
ABCA3					
CancerCode	pvalue	HR	Lower	Upper	Hazard Ratio(95%CI)
TCGA-STAD(N=372)	0.03	1.12	1.01	1.24	1.12(1.01,1.24)
TCGA-LUSC(N=468)	0.03	1.08	1.01	1.16	1.08(1.01,1.16)
TCGA-STES(N=547)	0.05	1.08	1.00	1.18	1.08(1.00,1.18)
TCGA-COADREAD(N=	0.0032	1.92	1.23	2.99	1.92(1.23,2.99)
TCGA-COAD(N=278)	0.06	1.59	0.97	2.60	1.59(0.97,2.60)

TCGA-PRAD(N=492)	0.08	2.88	0.83	10.05	2.88(0.83,10.05)
TCGA-UCEC(N=166)	0.05	2.16	0.97	4.80	2.16(0.97,4.80)
TCGA-BLCA(N=398)	0.14	1.26	0.93	1.72	1.26(0.93,1.72)
TCGA-GBMLGG(N=619	4E-27	0.57	0.51	0.63	0.57(0.51,0.63)
TCGA-LUAD(N=490)	0.00035	0.87	0.80	0.94	0.87(0.80,0.94)
TCGA-ACC(N=77)	0.0005	0.64	0.50	0.82	0.64(0.50,0.82)
TCGA-LGG(N=474)	0.0026	0.57	0.40	0.82	0.57(0.40,0.82)
TCGA-UVM(N=74)	0.0033	0.54	0.35	0.82	0.54(0.35,0.82)
TCGA-PAAD(N=172)	0.0077	0.77	0.64	0.93	0.77(0.64,0.93)
TCGA-KIRC(N=515)	0.01	0.79	0.65	0.95	0.79(0.65,0.95)
TCGA-SKCM-P(N=97)	0.01	0.70	0.53	0.93	0.70(0.53,0.93)
TCGA-CHOL(N=33)	0.02	0.60	0.38	0.92	0.60(0.38,0.92)
TCGA-KIPAN(N=855)	0.03	0.85	0.74	0.98	0.85(0.74,0.98)

ABCB1

CancerCode	pvalue	HR	Lower	Upper	Hazard Ratio(95%CI)
TCGA-LAML(N=209)	0.09	1.07	0.99	1.16	1.07(0.99,1.16)
TCGA-SKCM(N=444)	5.3E-07	0.80	0.73	0.87	0.80(0.73,0.87)
TCGA-SKCM-M(N=347	1.7E-06	0.79	0.72	0.87	0.79(0.72,0.87)
TCGA-GBMLGG(N=619	7.9E-06	0.69	0.59	0.81	0.69(0.59,0.81)
TCGA-KIRC(N=515)	0.00037	0.87	0.80	0.94	0.87(0.80,0.94)
TCGA-SARC(N=254)	0.0067	0.85	0.76	0.96	0.85(0.76,0.96)
TCGA-HNSC(N=509)	0.01	0.91	0.84	0.98	0.91(0.84,0.98)
TCGA-PAAD(N=172)	0.02	0.85	0.75	0.97	0.85(0.75,0.97)
TCGA-MESO(N=84)	0.04	0.85	0.73	0.99	0.85(0.73,0.99)
TCGA-BRCA(N=1044)	0.05	0.90	0.80	1.00	0.90(0.80,1.00)
TCGA-LUAD(N=490)	0.07	0.88	0.76	1.01	0.88(0.76,1.01)
TCGA-CESC(N=273)	0.09	0.85	0.71	1.02	0.85(0.71,1.02)
TCGA-OV(N=407)	0.13	0.92	0.83	1.02	0.92(0.83,1.02)

ABCC3

CancerCode	pvalue	HR	Lower	Upper	Hazard Ratio(95%CI)
TCGA-GBMLGG(N=619	3E-58	1.42	1.36	1.49	1.42(1.36,1.49)
TCGA-LGG(N=474)	1.5E-20	1.37	1.28	1.46	1.37(1.28,1.46)
TCGA-KIPAN(N=855)	4.4E-06	1.19	1.11	1.29	1.19(1.11,1.29)
TCGA-UVM(N=74)	0.0031	1.42	1.12	1.80	1.42(1.12,1.80)
TCGA-PAAD(N=172)	0.0037	1.24	1.07	1.43	1.24(1.07,1.43)
TCGA-KIRP(N=276)	0.02	1.30	1.05	1.61	1.30(1.05,1.61)
TCGA-HNSC(N=509)	0.02	1.13	1.02	1.26	1.13(1.02,1.26)
TCGA-GBM(N=144)	0.03	1.12	1.01	1.24	1.12(1.01,1.24)
TCGA-LIHC(N=341)	0.03	1.20	1.02	1.41	1.20(1.02,1.41)
TCGA-BLCA(N=398)	0.01	0.91	0.84	0.98	0.91(0.84,0.98)
TCGA-SKCM-M(N=347	0.02	0.90	0.82	0.98	0.90(0.82,0.98)
TCGA-SKCM(N=444)	0.03	0.91	0.84	0.99	0.91(0.84,0.99)
TCGA-BRCA(N=1044)	0.04	0.71	0.51	0.98	0.71(0.51,0.98)

ABCG1

CancerCode	pvalue	HR	Lower	Upper	Hazard Ratio(95%CI)
TCGA-STAD(N=372)	0.0051	1.26	1.07	1.47	1.26(1.07,1.47)
TCGA-STES(N=547)	0.01	1.18	1.04	1.35	1.18(1.04,1.35)
TCGA-LAML(N=209)	0.01	1.16	1.04	1.31	1.16(1.04,1.31)
TCGA-LIHC(N=341)	0.03	1.15	1.01	1.30	1.15(1.01,1.30)
TCGA-PRAD(N=492)	0.05	2.42	1.00	5.81	2.42(1.00,5.81)
TCGA-BRCA(N=1044)	0.05	1.41	0.99	2.01	1.41(0.99,2.01)
TCGA-GBMLGG(N=619	2.8E-13	0.58	0.50	0.67	0.58(0.50,0.67)
TCGA-KIRC(N=515)	1.6E-08	0.64	0.55	0.75	0.64(0.55,0.75)
TCGA-KIPAN(N=855)	0.000054	0.79	0.70	0.88	0.79(0.70,0.88)
TCGA-SKCM-M(N=347	0.0013	0.85	0.77	0.94	0.85(0.77,0.94)
TCGA-SKCM(N=444)	0.0017	0.86	0.79	0.95	0.86(0.79,0.95)
TARGET-ALL(N=86)	0.0025	0.76	0.63	0.91	0.76(0.63,0.91)
TCGA-LGG(N=474)	0.0031	0.70	0.55	0.89	0.70(0.55,0.89)

TCGA-KIRP(N=276)	0.02	0.75	0.58	0.96	0.75(0.58,0.96)
TCGA-CESC(N=273)	0.04	0.61	0.38	0.98	0.61(0.38,0.98)

ABCG2

CancerCode	pvalue	HR	Lower	Upper	Hazard Ratio(95%CI)
TCGA-BLCA(N=398)	0.07	1.09	0.99	1.19	1.09(0.99,1.19)
TCGA-BRCA(N=1044)	0.06	1.36	0.98	1.90	1.36(0.98,1.90)
TCGA-KIRC(N=515)	2.5E-13	0.70	0.64	0.77	0.70(0.64,0.77)
TCGA-GBMLGG(N=619	3.6E-08	0.65	0.55	0.75	0.65(0.55,0.75)
TCGA-KIPAN(N=855)	0.0084	0.91	0.85	0.98	0.91(0.85,0.98)
TCGA-GBM(N=144)	0.01	0.78	0.64	0.95	0.78(0.64,0.95)
TCGA-PAAD(N=172)	0.05	0.85	0.72	1.00	0.85(0.72,1.00)

ABCG4

CancerCode	pvalue	HR	Lower	Upper	Hazard Ratio(95%CI)
TCGA-STAD(N=372)	0.0015	1.25	1.09	1.43	1.25(1.09,1.43)
TCGA-MESO(N=84)	0.0057	1.23	1.06	1.42	1.23(1.06,1.42)
TCGA-STES(N=547)	0.02	1.12	1.02	1.24	1.12(1.02,1.24)
TCGA-SKCM(N=444)	0.02	1.10	1.02	1.19	1.10(1.02,1.19)
TCGA-UCEC(N=166)	0.03	1.22	1.02	1.47	1.22(1.02,1.47)
TCGA-BLCA(N=398)	0.03	1.11	1.01	1.22	1.11(1.01,1.22)
TCGA-BRCA(N=1044)	0.06	1.14	1.00	1.31	1.14(1.00,1.31)

ABCF2

CancerCode	pvalue	HR	Lower	Upper	Hazard Ratio(95%CI)
TCGA-GBMLGG(N=619	2.3E-12	2.77	2.09	3.68	2.77(2.09,3.68)
TCGA-LIHC(N=341)	0.00015	1.69	1.29	2.21	1.69(1.29,2.21)
TCGA-LGG(N=474)	0.0017	1.96	1.29	2.97	1.96(1.29,2.97)
TCGA-LAML(N=209)	0.0026	1.25	1.08	1.46	1.25(1.08,1.46)
TCGA-KICH(N=64)	0.0028	19.75	3.18	122.66	19.75(3.18,122.66)
TCGA-BLCA(N=398)	0.0084	1.39	1.09	1.77	1.39(1.09,1.77)
TCGA-LUAD(N=490)	0.0053	1.53	1.13	2.06	1.53(1.13,2.06)
TCGA-BRCA(N=1044)	0.02	1.54	1.05	2.26	1.54(1.05,2.26)
TCGA-KIRC(N=515)	0.05	0.77	0.59	1.00	0.77(0.59,1.00)

MMP9

CancerCode	pvalue	HR	Lower	Upper	Hazard Ratio(95%CI)
TCGA-GBMLGG(N=619	1.4E-41	1.28	1.23	1.33	1.28(1.23,1.33)
TCGA-KIPAN(N=855)	4.5E-06	1.15	1.08	1.22	1.15(1.08,1.22)
TCGA-UVM(N=74)	7.4E-06	1.63	1.32	2.02	1.63(1.32,2.02)
TCGA-LGG(N=474)	0.000028	1.14	1.07	1.22	1.14(1.07,1.22)
TCGA-ACC(N=77)	0.0004	1.38	1.15	1.66	1.38(1.15,1.66)
TCGA-KIRC(N=515)	0.00047	1.14	1.06	1.22	1.14(1.06,1.22)
TCGA-LIHC(N=341)	0.0059	1.11	1.03	1.20	1.11(1.03,1.20)
TCGA-BLCA(N=398)	0.0075	1.08	1.02	1.15	1.08(1.02,1.15)
TCGA-TGCT(N=128)	0.03	2.98	1.04	8.51	2.98(1.04,8.51)
TCGA-PAAD(N=172)	0.07	1.11	0.99	1.24	1.11(0.99,1.24)
TCGA-SKCM(N=444)	0.02	0.94	0.89	0.99	0.94(0.89,0.99)
TCGA-SKCM-M(N=347	0.04	0.94	0.89	1.00	0.94(0.89,1.00)

SNAI1

CancerCode	pvalue	HR	Lower	Upper	Hazard Ratio(95%CI)
TCGA-GBMLGG(N=619	2.4E-29	1.51	1.41	1.63	1.51(1.41,1.63)
TCGA-LGG(N=474)	3E-08	1.39	1.23	1.56	1.39(1.23,1.56)
TCGA-KIPAN(N=855)	0.000007	1.19	1.10	1.29	1.19(1.10,1.29)
TCGA-THCA(N=501)	0.000019	2.45	1.64	3.65	2.45(1.64,3.65)
TCGA-LUSC(N=468)	0.0039	1.20	1.06	1.36	1.20(1.06,1.36)
TCGA-STES(N=547)	0.0043	1.19	1.06	1.34	1.19(1.06,1.34)
TCGA-KIRP(N=276)	0.0053	1.28	1.08	1.52	1.28(1.08,1.52)
TCGA-BLCA(N=398)	0.0075	1.15	1.04	1.27	1.15(1.04,1.27)
TCGA-BRCA(N=1044)	0.0074	1.80	1.16	2.79	1.80(1.16,2.79)
TCGA-LUAD(N=490)	0.02	1.16	1.03	1.31	1.16(1.03,1.31)
TCGA-STAD(N=372)	0.02	1.21	1.03	1.41	1.21(1.03,1.41)

TCGA-HNSC(N=509)	0.02	1.14	1.02	1.27	1.14(1.02,1.27)
TCGA-CESC(N=273)	0.03	1.19	1.02	1.39	1.19(1.02,1.39)
TCGA-COADREAD(N=	0.03	1.29	1.02	1.62	1.29(1.02,1.62)
TCGA-COAD(N=278)	0.04	1.31	1.01	1.69	1.31(1.01,1.69)
SNAI2					
CancerCode	pvalue	HR	Lower	Upper	Hazard Ratio(95%CI)
TCGA-GBMLGG(N=61	4.8E-41	1.71	1.58	1.86	1.71(1.58,1.86)
TCGA-LGG(N=474)	2.3E-10	1.57	1.36	1.80	1.57(1.36,1.80)
TCGA-KIPAN(N=855)	1.7E-06	1.20	1.12	1.30	1.20(1.12,1.30)
TCGA-LUAD(N=490)	0.00033	1.23	1.10	1.37	1.23(1.10,1.37)
TCGA-MESO(N=84)	0.00055	1.39	1.15	1.68	1.39(1.15,1.68)
TCGA-PAAD(N=172)	0.0041	1.25	1.07	1.45	1.25(1.07,1.45)
TCGA-KIRP(N=276)	0.0059	1.28	1.07	1.52	1.28(1.07,1.52)
TCGA-STAD(N=372)	0.0075	1.20	1.05	1.37	1.20(1.05,1.37)
TCGA-ACC(N=77)	0.0078	1.41	1.09	1.82	1.41(1.09,1.82)
TCGA-KICH(N=64)	0.01	1.96	1.17	3.30	1.96(1.17,3.30)
TCGA-SKCM(N=444)	0.03	1.11	1.01	1.22	1.11(1.01,1.22)
TCGA-BLCA(N=398)	0.03	1.11	1.01	1.21	1.11(1.01,1.21)
SNAI3					
CancerCode	pvalue	HR	Lower	Upper	Hazard Ratio(95%CI)
TCGA-LAML(N=209)	0.0054	1.20	1.05	1.36	1.20(1.05,1.36)
TCGA-COADREAD(N=	0.06	1.19	0.99	1.42	1.19(0.99,1.42)
TCGA-LUAD(N=490)	0.00039	0.77	0.67	0.89	0.77(0.67,0.89)
TCGA-CESC(N=273)	0.0015	0.72	0.59	0.88	0.72(0.59,0.88)
TCGA-SKCM(N=444)	0.0037	0.87	0.79	0.96	0.87(0.79,0.96)
TCGA-PAAD(N=172)	0.01	0.79	0.65	0.95	0.79(0.65,0.95)
TCGA-SARC(N=254)	0.03	0.82	0.68	0.98	0.82(0.68,0.98)
TCGA-SKCM-M(N=347	0.05	0.90	0.81	1.00	0.90(0.81,1.00)
TCGA-SKCM-P(N=97)	0.06	0.72	0.51	1.02	0.72(0.51,1.02)
