# Supplementary material for: A comprehensive landscape analysis of autophagy in cancer development and drug resistance
Source: Front Immunol. 2024 Aug 26;15:1412781. doi: 10.3389/fimmu.2024.1412781 (PMC11381251; doi:10.3389/fimmu.2024.1412781)
Supplement: Supplementary file 3 [file DataSheet3.docx]

**Table S3 The correlation analysis between autophagy-associated genes (ULK1, ULK2, ATG7, CDKN1A; CAMKK2; GABARAPL1; GABARAPL2) and tumor resistance/metastasis-associated genes.**

| Gene | ABCA2 | ABCA3 | ABCB1 | ABCC3 | ABCG1 | ABCG2 | ABCG4 | ABCF2 | MMP9 | SNAI1 | SNAI2 | SNAI3 |
| --- | --- | --- | --- | --- | --- | --- | --- | --- | --- | --- | --- | --- |
| ULK1 | BRCA,0.48；  LUSC, 0.54;  PAAD, 0.53;  GBM, 0.59;  PRAD, 0.61;  DLBC, 0.79 | BRCA,0.32；  OV, 0.54;  THCA, 0.46 | DLBC,0.53;  STAD, 0.33;  THCA,0.38; | CHOL,0.29;  ESCA, -0.38;  THCA, 0.26 | THCA,0.50;  BRCA,0.27;  LIHC, 0.30; | DLBC,0.44;  THCA,0.32; | UCEC,0.37;  STAD,0.38 | OV,0.31;  PRAD,0.42;  THCA,0.49;  DLBC,0.64;  LIHC,0.43; | COAD,0.31; | THCA,0.40;  STAD,0.30 | ESCA,0.52;  STAD,0.21 | STAD,0.34;  LIHC,0.24; |
| ULK2 | BRCA,0.38  PRAD, 0.57;  PAAD, 0.44;  STAD, 0.39;  THCA, 0.43; | BRCA, 0.44;  KIRC, 0.47;  LIHC, 0.39;  PAAD, 0.48;  PRAD, 0.49;  THCA, 0.44;  STAD, 0.50 | THCA, 0.27;  STAD, 0.43;  PRAD, 0.41;  KIRP,0.36;  KIRC, 0.33; | ESCA, -0.30;  PAAD, -0.28 | THCA, 0.31;  STAD,0.37 | THCA, 0.35;  PRAD,0.40;  PAAD,0.61  KIRC,0.32;  HNSC, 0.35;  BRCA,0.28 | LGG, 0.27;  STAD,0.37 | BRCA, 0.25;  ESCA,0.32;  KIRC, 0.41;  KIRP,0.46;  PAAD,0.42;  PRAD,0.53;  THCA, 0.67 |  | DLBC, 0.54;  LIHC, 0.30;  PAAD,0.43; | STAD,0.40;  PAAD,0.36;  HNSC, 0.29  ESCA, 0.50 | STAD, 0.24 |
| ATG7 | HNSC,0.29;  STAD,0.34; | LUSC,0.27;  LIHC,0.32;  KIRP, 0.35 | BRCA,0.21;  ESCA, 0.34;  LUSC,0.37;  STAD,0.33; | CESC,0.29; | OV, 0.30;  KIRP, 0.37;  LIHC, 0.30;  PRAD,0.30;  STAD,0.40 |  |  | KIRP, 0.45;  KIRC,0.37;  LIHC,0.45;  PAAD, 0.32;  STAD, 0.36;  THCA, 0.37 | KIRP, 0.35;  LIHC, 0.48;  LUAD, 0.39;  LUSC, 0.32;  PAAD,0.37;  STAD,0.36;  THCA, 0.35 | BLCA, 0.32;  LUSC,0.37;  PAAD, 0.34; | KIRC,0.34;  LUAD, 0.36;  PAAD,0.48; | KIRP,0.38;  LIHC,0.41;  LUSC, 0.40; |
| CDKN1A | LIHC, 0.35; | KIRP,0.38 | PRAD,0.31; |  |  |  |  |  | LUSC,0.29 | KIRC,0.30;  LIHC,0.3;  LUAD, 0.34;  PAAD, 0.39; | OV, 0.45;  LUAD,0.33;  BLCA,0.34 |  |
| CAMKK2 | BRCA,0.36;  CESC,0.32;  KIRC,0.51;  KIRP, 0.36;  LIHC,0.41;  STAD, 0.45 | BRCA, 0.37;  KIRC,0.48;  PRAD, 0.40;  STAD,0.35;  THCA, 0.45 | KIRP, 0.5;  STAD, 0.36;  THCA, 0.36 |  | BRCA,0.26;  KIRC, 0.30;  STAD,0.32; | KIRC, 0.26;  PAAD, 0.38;  THCA, 0.31; | LUAD, 0.30;  PAAD, 0.32; | BLCA, 0.42;  BRCA, 0.37;  CESC, 0.47;  COAD, 0.40;  ESCA, 0.43;  KIRC, 0.39;  LIHC, 0.48;  PAAD, 0.50;  OV, 0.40;  STAD, 0.50;  THCA, 0.57 | PAAD, 0.28; | PAAD, 0.35;  THCA, 0.37 | COAD, 0.26;  KIRC, 0.36;  PAAD, 0.42;  PRAD, -0.29;  STAD, 0.29;  UCEC, 0.30 | STAD, 0.30 |
| *GABARAPL1* | ESCA, 0.36;  HNSC, 0.48;  LUSC, 0.31;  PAAD, 0.30;  THCA,0.42; | PAAD, 0.32;  STAD, 0.33;  THCA, 0.36 | BLCA, 0.28;  BRCA, 0.35;  HNSC, 0.32;  PAAD, 0.52;  PRAD, 0.42;  STAD, 0.41;  THCA, 0.35; | LUSC, 0.29; | ESCA, 0.34;  HNSC, 0.36;  KIRP, 0.31;  LUSC, 0.33; | BRCA, 0.31;  LIHC, 0.45;  PAAD, 0.38;  PRAD, 0.33;  THCA, 0.41 | COAD, 0.29;  PAAD, 0.36;  STAD, 0.49; | KIRC, 0.37;  THCA, 0.47; | LIHC, -0.33; | BLCA, 0.30;  HNSC, 0.39;  PAAD, 0.39;  PRAD, 0.35;  STAD, 0.31; | BRCA, 0.42;  COAD, 0.54;  ESCA, 0.38;  PRAD, 0.38;  STAD, 0.45; | COAD, 0.31; |
| *GABARAPL2* | LIHC, 0.28;  PAAD, 0.52;  THCA, 0.48; | KIRP, 0.36;  PAAD, 0.37; | PAAD, 0.29; | KIRC, -0.28; | PAAD, 0.31; | KIRC, 0.44;  LIHC, 0.33;  THCA, 0.37 |  | KIRC, 0.31;  KIRP, 0.35;  LIHC, 0.42;  UCEC, 0.32 | KIRC, -0.31;  THCA, -0.28; |  | LIHC, 0.26;  UCEC, 0.29; |  |

The correlation analysis between autophagy-associated genes (ULK1, ULK2, ATG7, CDKN1A; CAMKK2; GABARAPL1; GABARAPL2) and tumor resistance/metastasis-associated genes (ABCA2; ABCA3; ABCB1; ABCC3; ABCG1; ABCG2; ABCG4; ABCF2/MMP9; SNAI1; SNAI2; SNAI3) in pan-cancer from TCGA datasets, together with the Spearman’s rho value (all p<0.001). We only reported the correlation in some tumor types with significant significance and high degree of correlation.
