# Supplementary figures and images for: A comprehensive landscape analysis of autophagy in cancer development and drug resistance
Source: Front Immunol. 2024 Aug 26;15:1412781. doi: 10.3389/fimmu.2024.1412781 (PMC11381251; doi:10.3389/fimmu.2024.1412781)

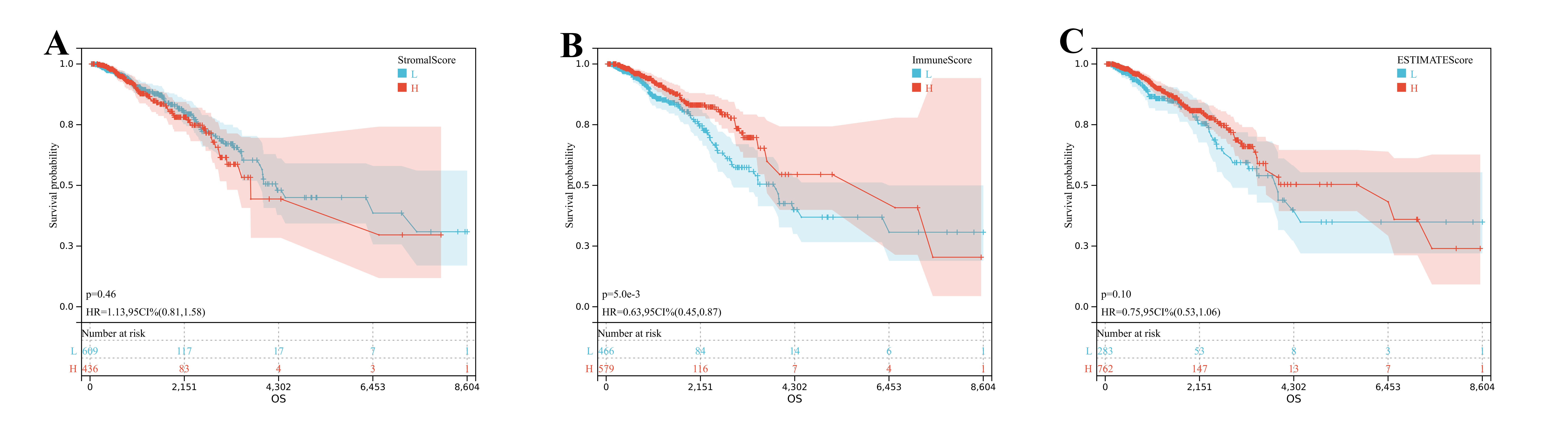

Supplement: Supplementary file 4 [file Image1.jpg]
